# Supplementary material for: Nanoscale and functional heterogeneity of the hippocampal extracellular space
Source: Cell Rep. 2023 May 5;42(5):112478. doi: 10.1016/j.celrep.2023.112478 (PMC10242443; doi:10.1016/j.celrep.2023.112478)
Supplement: Document S1. Figures S1–S10 [file mmc1.pdf]

Cell Reports, Volume *42*

Supplemental information

Nanoscale and functional heterogeneity

# of the hippocampal extracellular space

Diego Grassi, Agata Idziak, Antony Lee, Ivo Calaresu, Jean-Baptiste Sibarita, Laurent Cognet, U. Valentin Nägerl, and Laurent Groc

Suppl. Figure 1

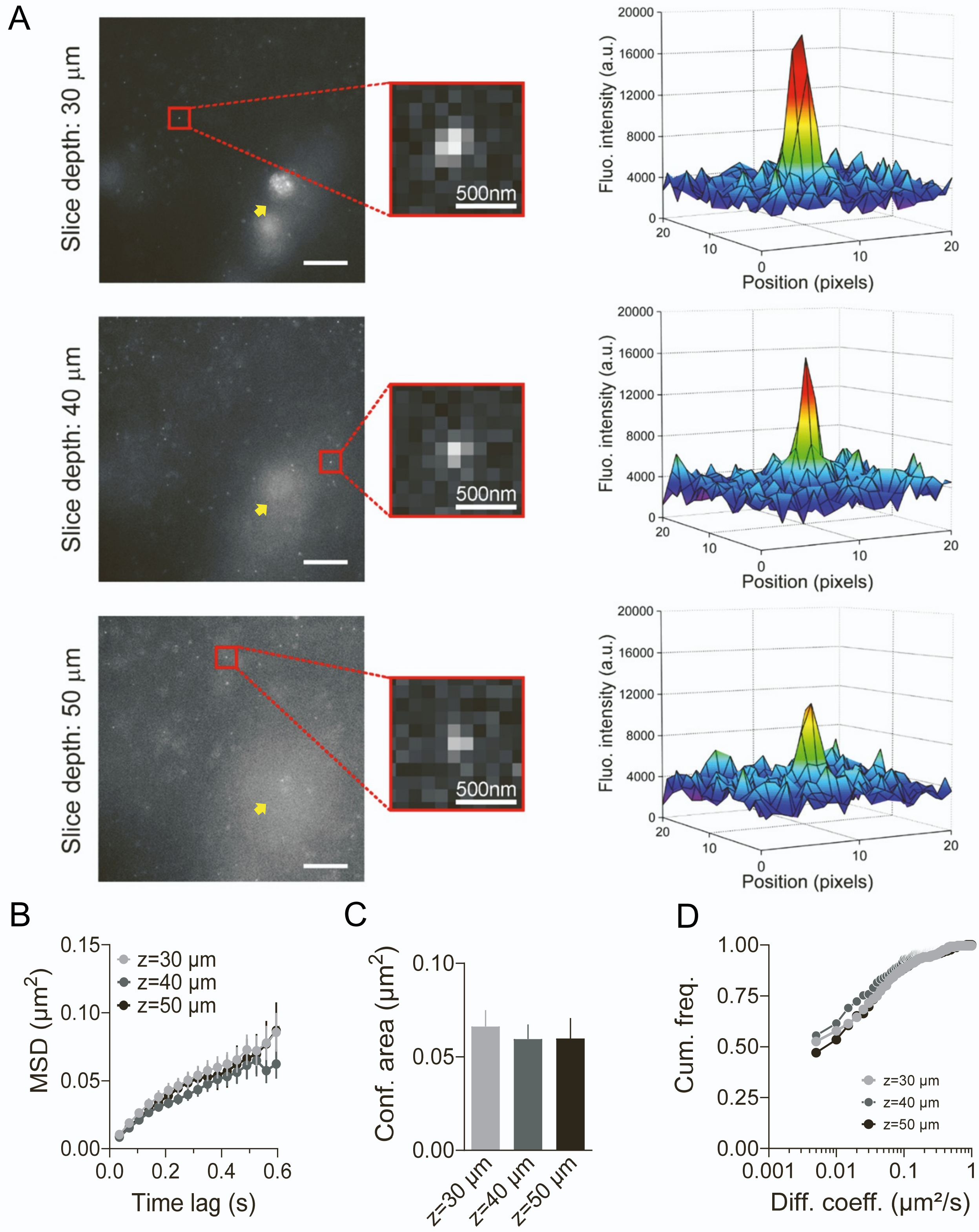

Figure S1. QD detection in hippocampal slice (Related to Figure 1)

(A) Left panles: QDs imaged at different depths (30, 40 and 50 µm) on a brain hippocampal slice (scale bars = 15 µm). Yellow arrows indicate the position of a cell body taken as reference. Red squares and enlarged insets show individual QDs visualized at different depths (scale bars, 500 nm). Right panels: heat map representation of fluorescence intensity of QDs indicated by red squares at different depths.

It should be noted that the QD tracking assays are usually performed at a depth of 30-50 µm since at depth below 50 µm the signal starts to exhibit significant degradation.

(B) MSD versus time plots for QD detections at different depths.

(C) Confinement areas represented by the mean ± SEM values for QD detections at different depths. Confinement areas were calculated based on their corresponding MSD curves.

(D) Diffusion coefficient cumulative distributions for QD recorded at different depths.

Suppl. Figure 2

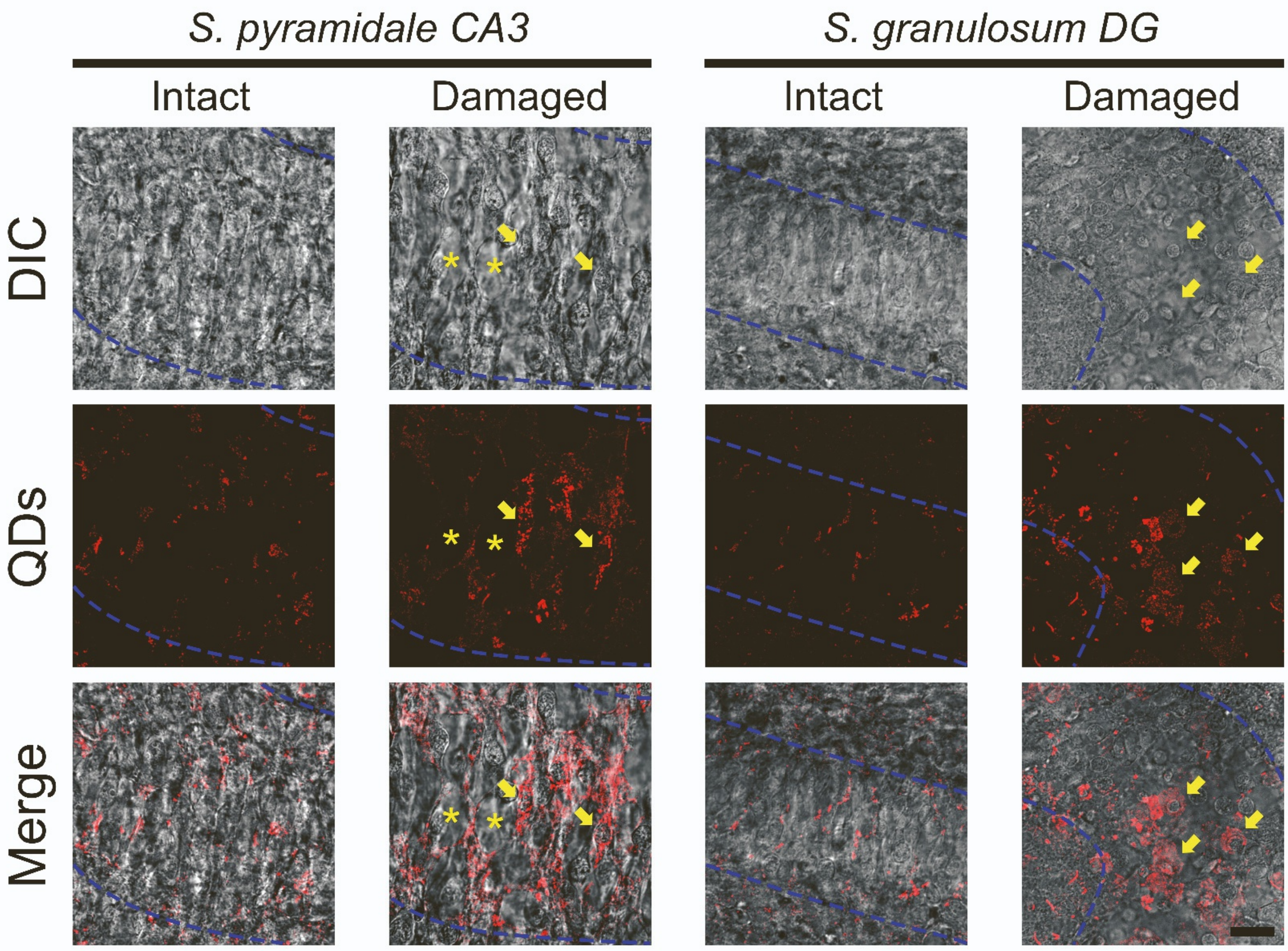

Figure S2. Quantum dot dispersion in intact or damaged brain tissue (Related to Figure 1)
Distribution of quantum dots in intact (first and third columns) or damaged (second and forth columns) tissue on DIV 7 organotypic brain slices (gray, DIC micrographies; red, quantum dots maximal projections of 500 frames). Cell layers of *s. pyramidale* CA3 and *s. granulosum* DG are delimited within blue dashed lines, yellow asterisks indicate cells exhibiting considerable swelling and yellow arrows mark dead cells with quantum dots invading the cytoplasm. Scale bar = 15 µm.

Suppl. Figure 3

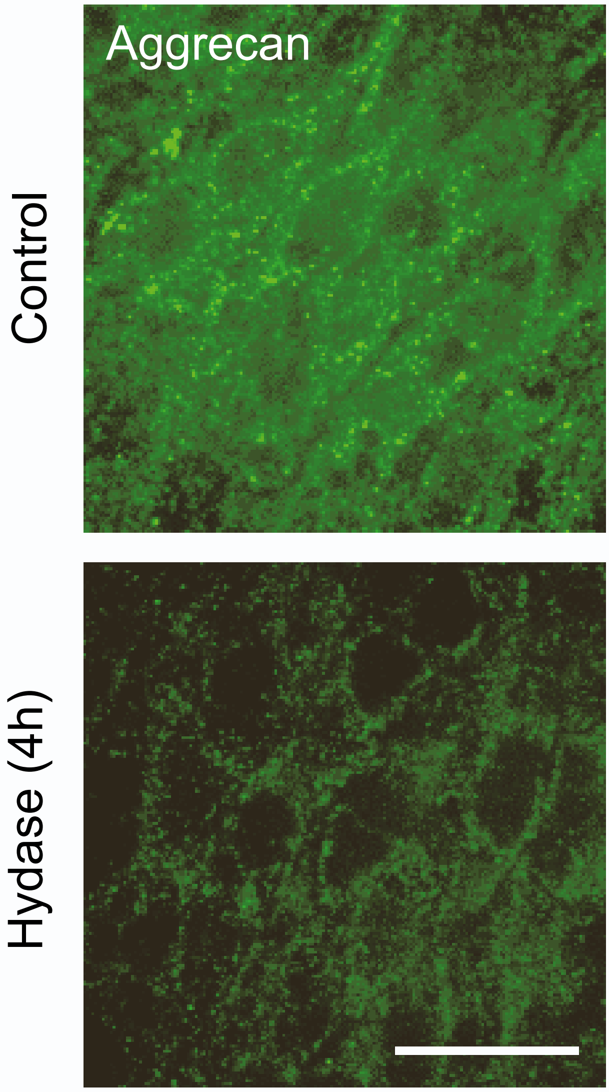

Figure S3. Effect of hyaluronidase on hippocampal ECM (Related to Figures 1 and 2)
Representative images of aggrecan immunostaining in control, untreated (upper panel) and hyaluronidase-treated (lower panel) CA3 hippocampal area (scale bar = 25 µm).

Suppl. Figure 4

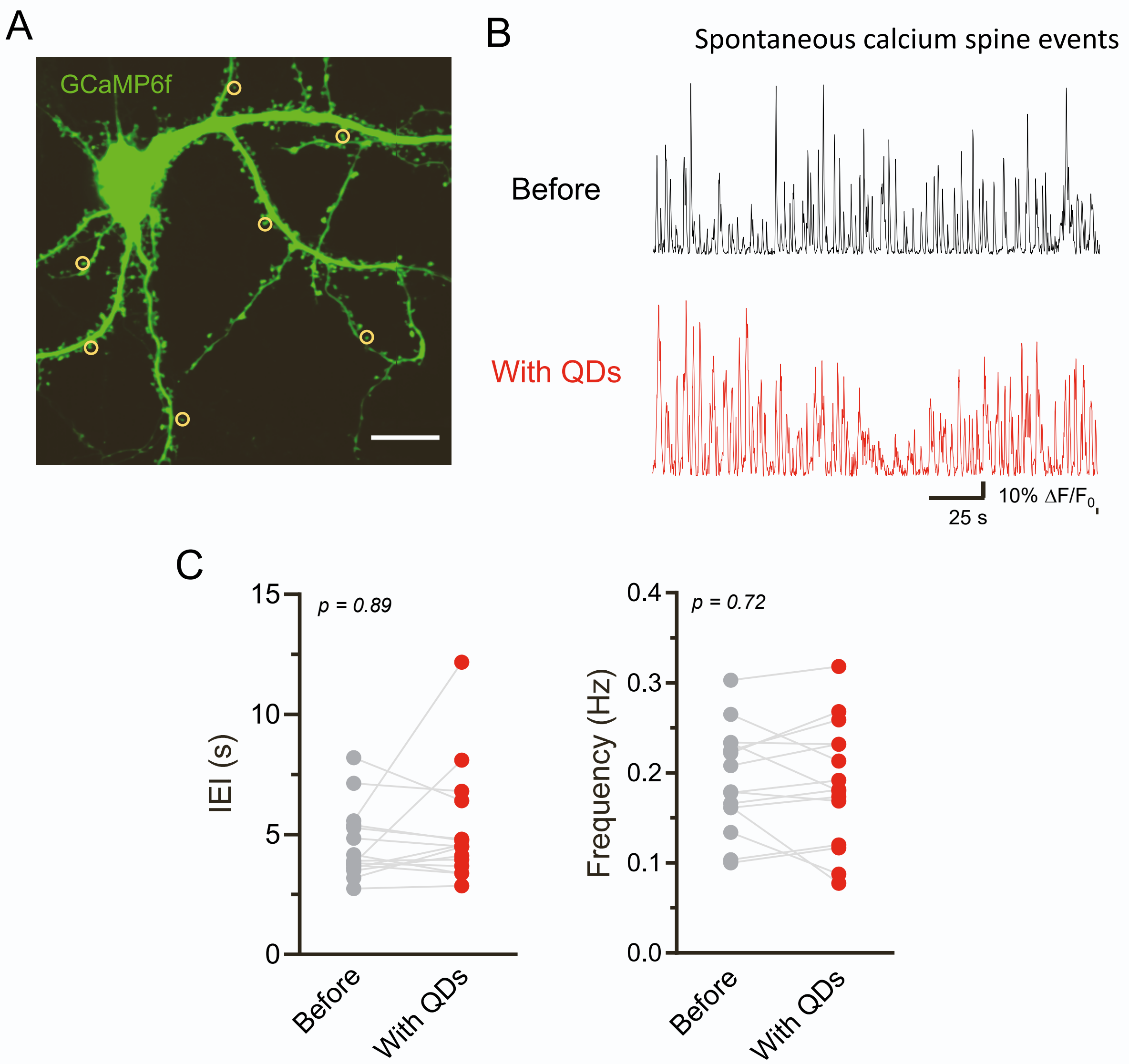

Figure S4. Neuronal calcium dynamics recording from dendritic spines upon acute QD exposure (Related to Figure 1)
(A) Snapshot of a representative field of view of a hippocampal neuron expressing GCaMP6f Ca2+ reporter is shown together with circular ROIs (yellow) around spines from which activity was monitored (Scale bar = 15 μm).
(B) Representative time series of Ca2+ transients recorded pre and post QD incubation are also provided (upper right). Calcium transients are expressed as fractional amplitude increase.
(C) Before and after plots illustrate averaged values of spines activity expressed as both inter event intervals (left) and frequency (right) for each recorded neuron. As denoted by p-values, under these experimental conditions no significative differences have been observed pre and post QD exposure.

Suppl. Figure 5

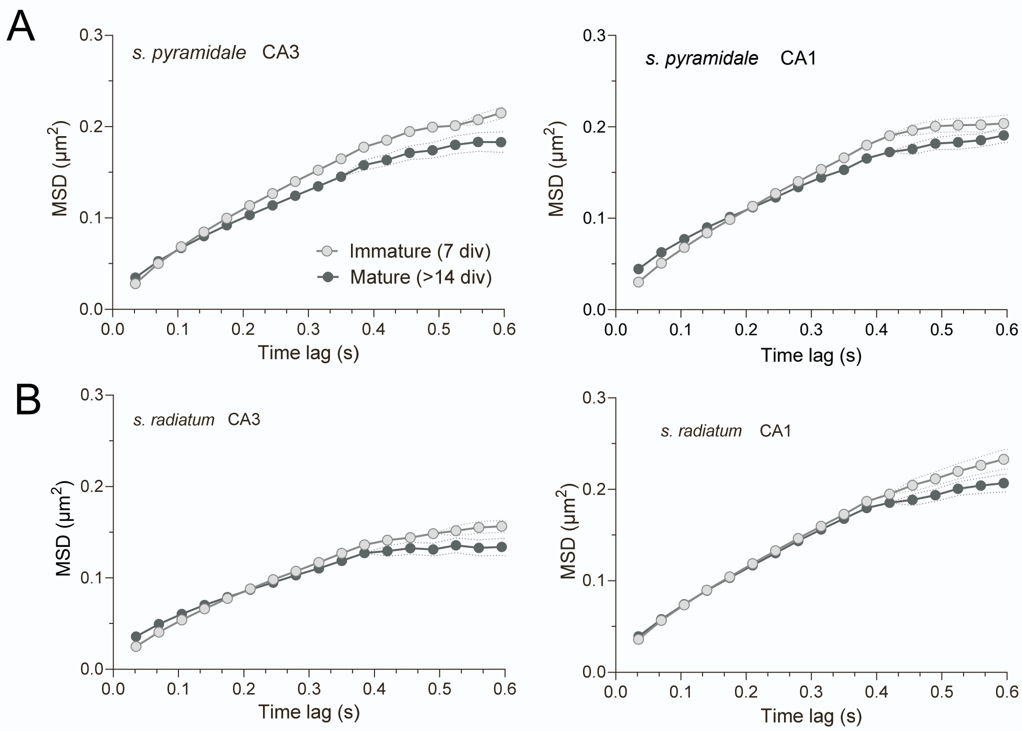

Figure S5. Comparison of QD-based ECS measurements at different developmental stages (Related to Figure 1)
(A) MSD versus time plots for *s. pyramidale* in CA3 (left panel) and CA1 (right panel) areas at 7 and >14 DIV.
(B) MSD versus time plots for *s. radiatum* in CA3 (left panel) and CA1 (right panel) areas at 7 and >14 DIV.

Suppl. Figure 6

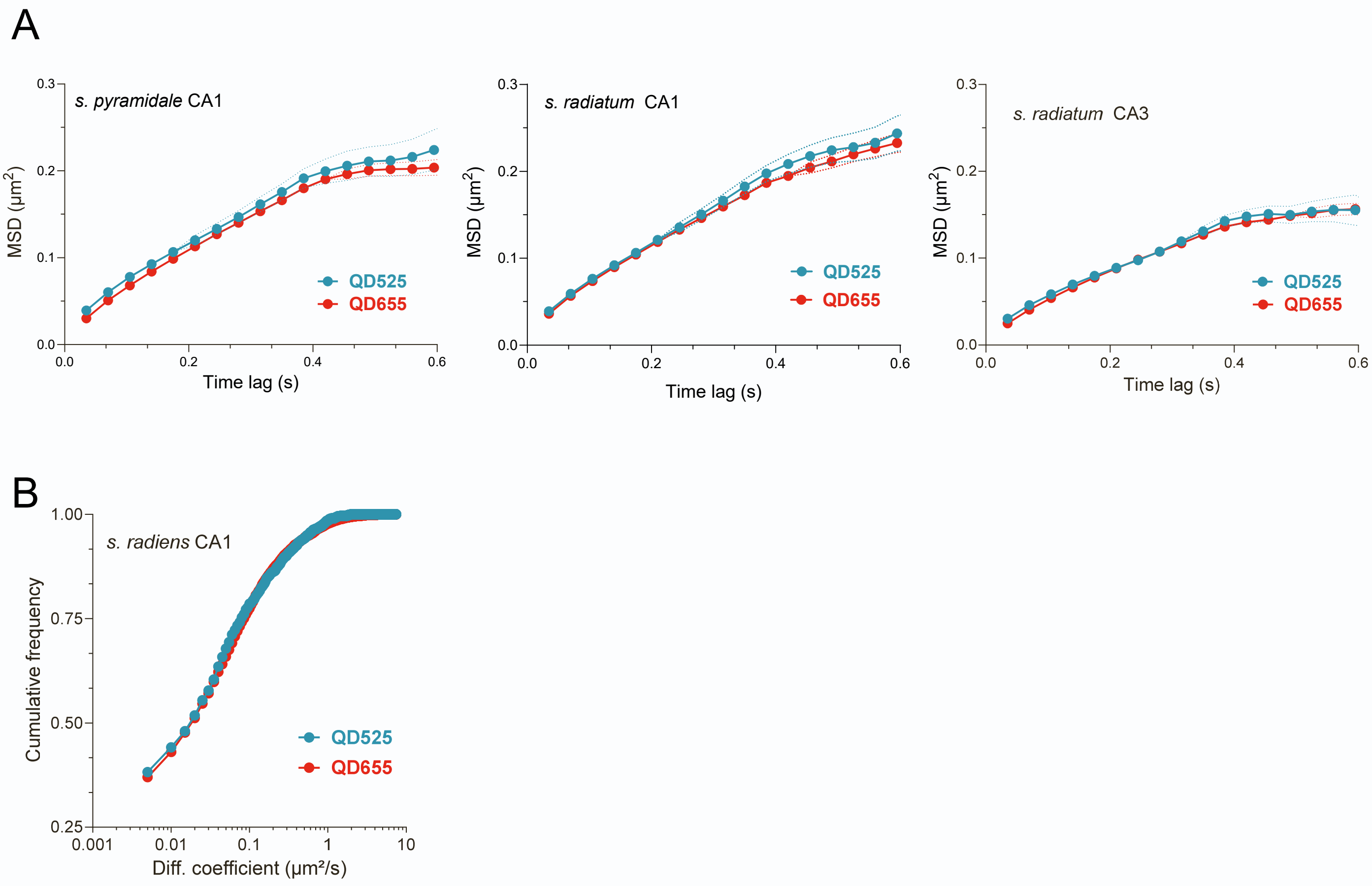

Figure S6. Comparison of QD-based ECS measurements from two different QD species (Related to Figure 1)
(A) MSD versus time plots of QD525 and QD655 for *s. pyramidale* in CA1 (left panel), *s. radiatum* in CA1 (middle panel) and *s. radiatum* in CA3 (right panel) areas.
(B) Diffusion coefficient cumulative distributions of QD525 and QD655 for s. *radiatum* in CA1 area.

Suppl. Figure 7

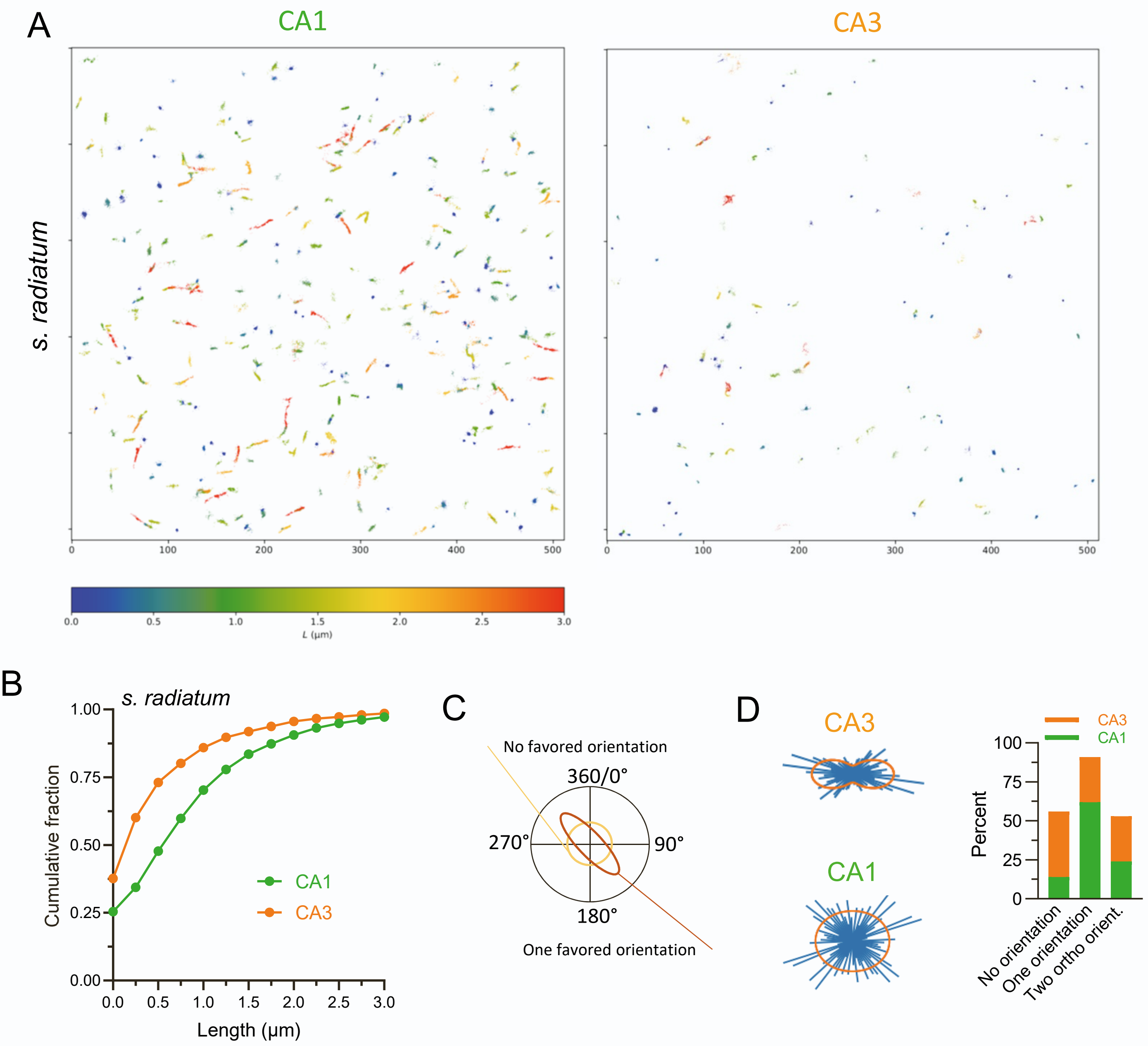

Figure S7. QD-based objects measurements (Related to Figure 1)
(A) Representative images showing length variations for s. *radiatum* in CA1 (left panel) and CA3 (right panel) areas using a QD-based cloud points analysis. Color-graded scale bar.
(B) Cumulative fractions plot of measured lengths for s. radiatum in CA3 and CA1 areas using a QD-based cloud point analysis.

Suppl. Figure 8

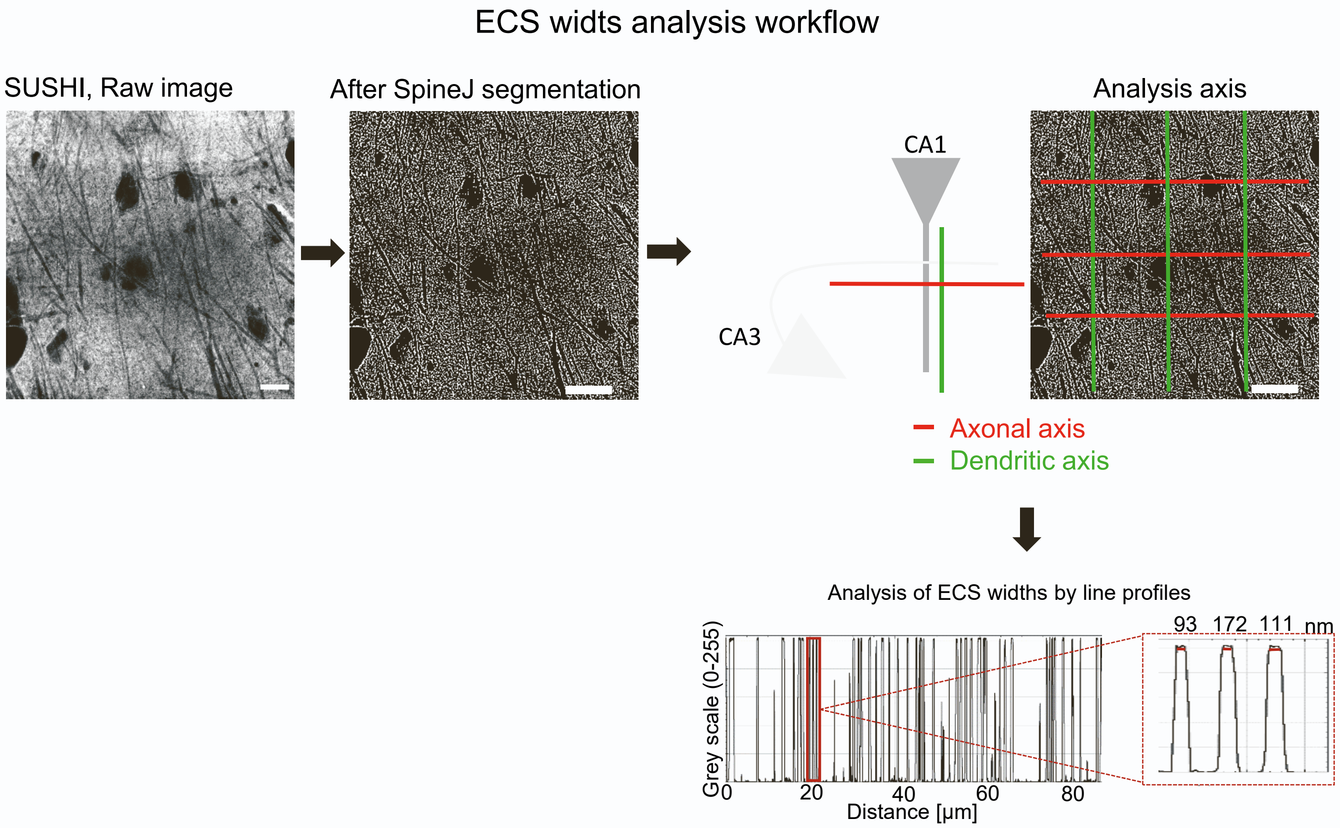

Figure S8. SUSHI-based ECS width analysis workflow (Related to Figure 3)
ECS widths analysis workflow. *Left panel*, Raw SUSHI image showing the ECS (white) and cellular elements (black) for a section of *s. radiatum* in the CA1 area (scale bar = 10 µm). *Middle panel*, A wavelet – based algorithm is used to binarize the raw SUSHI images. *Right panel*, Simplistic drawing of the CA3-CA1 connectivity, highliting the dendritic (green) and axonal (red) axis in the CA1 *s. radiatum*. Scanlines (line profiles) are drawn following the dendritic axis (i.e., paralell to the dendrites, green) and axonal axis (i.e., parallel to Schaffer collateral axons, red). *Low panel*: The peaks detected along the scanlines are measured to extract the ECS width on each detection, and then these values are plotted according their sizes.

Suppl. Figure 9

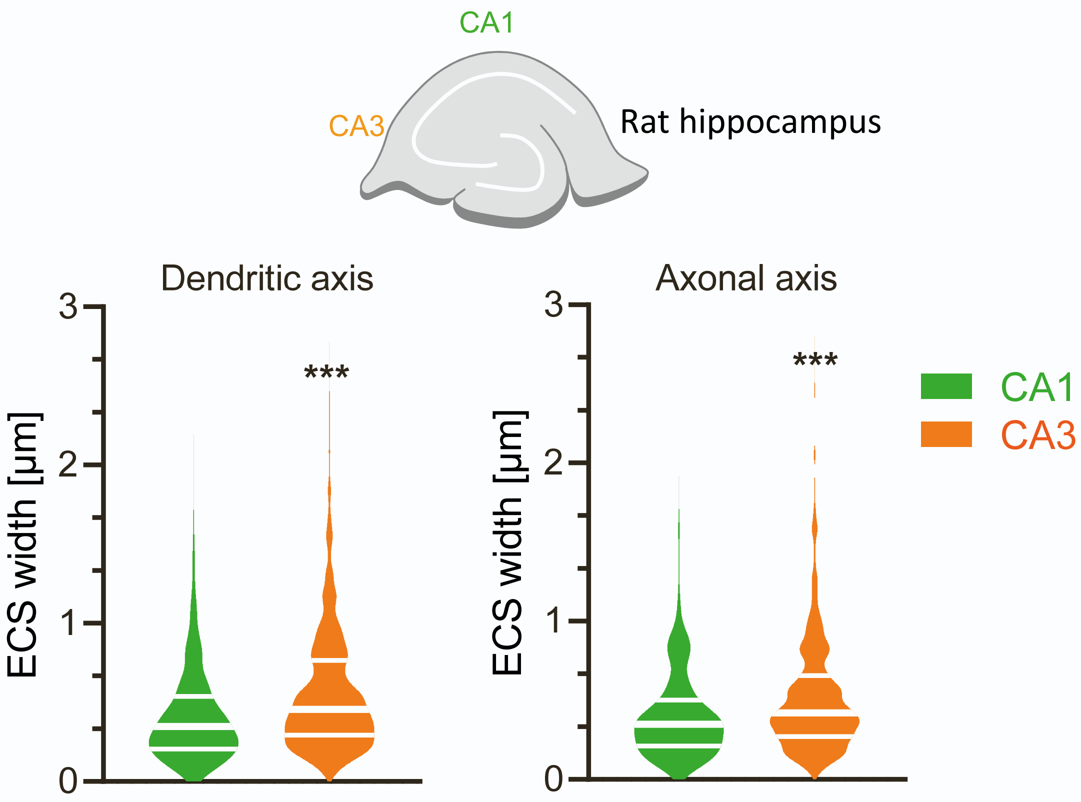

Figure S9. SUSHI-based exploration of the rat CA1 and CA3 areas (Related to Figure 3)
(A) Schematic representation of a rat hippocampal slice depicting CA3 and CA1 areas.
(B) ECS widths plots for *s. radiatum* in CA1 and CA3 areas for the dendritic axis (i.e., parallel to the dendrites) and axonal axis (i.e., parallel to Schaffer collateral axons).

Suppl. Figure 10

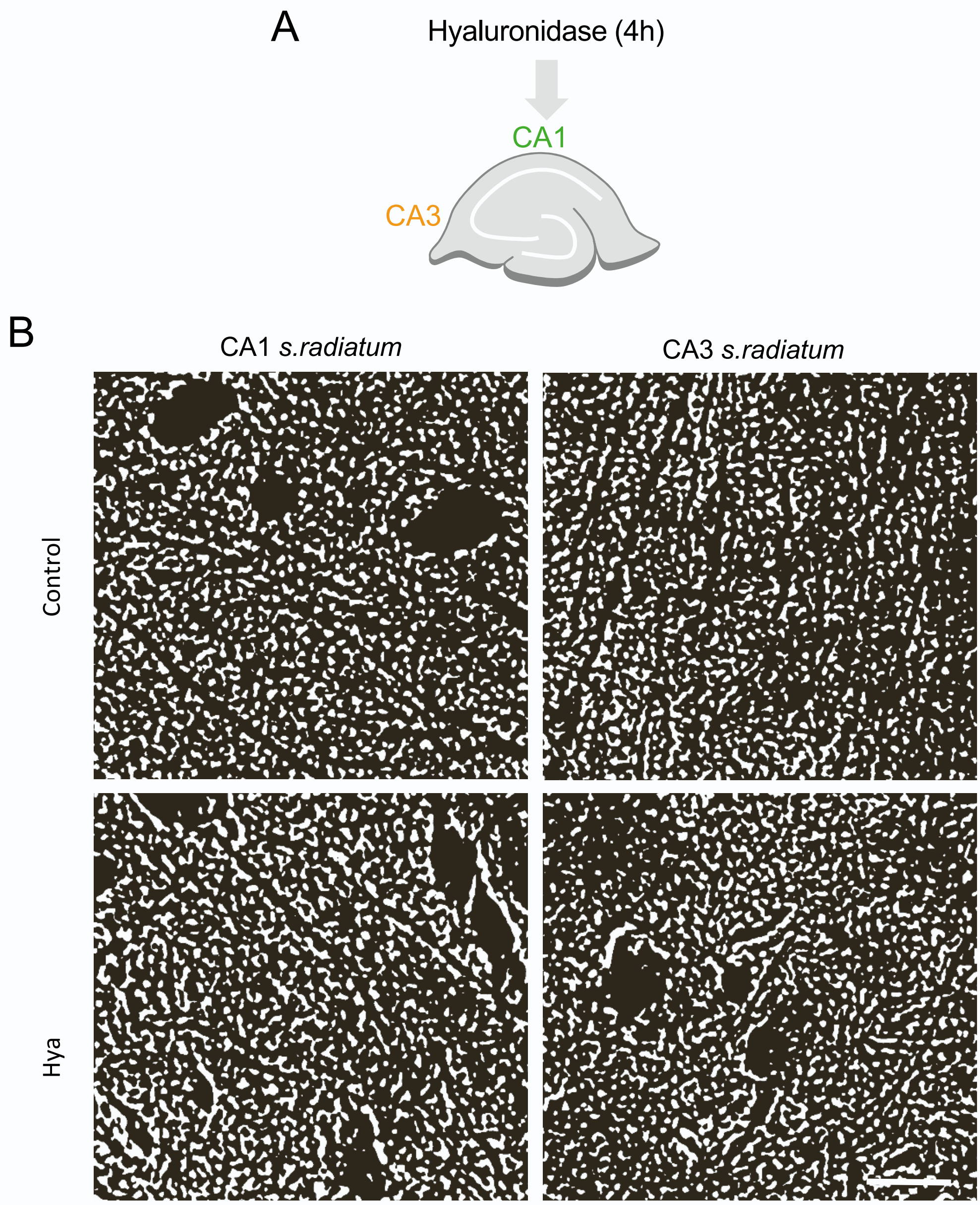

Figure S10. SUSHI-based imaging of CA1 and CA3 areas exposed to hyaluronidase (Related to Figure 3)
(A) Schematic representation of a rat hippocampal slice depicting CA3 and CA1 areas exposed to 4h treatment with hyaluronidase.
(B) Raw SUSHI images showing the ECS (white) for a section of *s. radiatum* in the CA1 and CA3 areas exposed to hyaluronidase (scale bar = 20 µm).
