# Supplementary material for: Nanoscale and functional heterogeneity of the hippocampal extracellular space
Source: Cell Rep. 2023 May 5;42(5):112478. doi: 10.1016/j.celrep.2023.112478 (PMC10242443; doi:10.1016/j.celrep.2023.112478)
Supplement: Document S2. Article plus supplemental information [file mmc2.pdf]

Cell Reports

Report

Nanoscale and functional heterogeneity of the hippocampal extracellular space

Graphical abstract

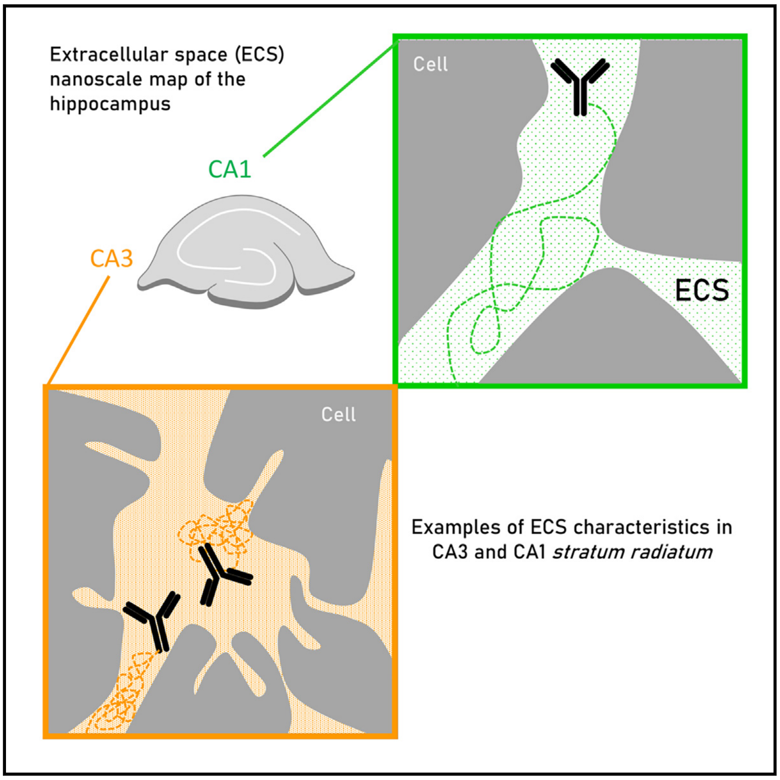

Highlights

- Nanoscale map of the hippocampal formation extracellular space
- The extracellular space characteristics differ between areas and layers
- The CA1 *stratum radiatum* extracellular space is smaller but more fluid than in CA3
- The extracellular space characteristics tune immunoglobulin distribution

Authors

Diego Grassi, Agata Idziak, Antony Lee, ..., Laurent Cognet, U. Valentin Nägerl, Laurent Groc

Correspondence

laurent.groc@u-bordeaux.fr

In brief

Grassi et al. describe the nanoscale organization of the extracellular space of the rodent hippocampus. Its physical properties differ strongly across the hippocampus, in particular between the *stratum radiatum* of CA1/3. These marked differences go hand in hand with differences in the dynamics and distribution of immunoglobulins in these areas.

Grassi et al., 2023, Cell Reports *42*, 112478
May 30, 2023 © 2023 The Author(s).
https://doi.org/10.1016/j.celrep.2023.112478

Cell Reports

CellPress
OPEN ACCESS

Report

Nanoscale and functional heterogeneity of the hippocampal extracellular space

Diego Grassi,[1] Agata Idziak,[1,4] Antony Lee,[2,3,4] Ivo Calaresu,[1] Jean-Baptiste Sibarita,[1] Laurent Cognet,[2,3] U. Valentin Nägerl,[1] and Laurent Groc[1,5,*]
[1]University of Bordeaux, CNRS, Interdisciplinary Institute for Neuroscience, IINS, UMR 5297, 33000 Bordeaux, France
[2]University of Bordeaux, Laboratoire Photonique Numérique et Nanosciences (LP2N), UMR 5298, 33400 Talence, France
[3]Institut d'Optique & CNRS, LP2N UMR 5298, 33400 Talence, France
[4]These authors contributed equally
[5]Lead contact
*Correspondence: laurent.groc@u-bordeaux.fr
https://doi.org/10.1016/j.celrep.2023.112478

SUMMARY

The extracellular space (ECS) and its constituents play a crucial role in brain development, plasticity, circadian rhythm, and behavior, as well as brain diseases. Yet, since this compartment has an intricate geometry and nanoscale dimensions, its detailed exploration in live tissue has remained an unmet challenge. Here, we used a combination of single-nanoparticle tracking and super-resolution microscopy approaches to map the nanoscale dimensions of the ECS across the rodent hippocampus. We report that these dimensions are heterogeneous between hippocampal areas. Notably, *stratum radiatum* CA1 and CA3 ECS differ in several characteristics, a difference that gets abolished after digestion of the extracellular matrix. The dynamics of extracellular immunoglobulins vary within these areas, consistent with their distinct ECS characteristics. Altogether, we demonstrate that ECS nanoscale anatomy and diffusion properties are widely heterogeneous across hippocampal areas, impacting the dynamics and distribution of extracellular molecules.

INTRODUCTION

The cerebral tissue is composed of cells, such as neurons and astrocytes, that are separated by an extracellular space (ECS) in which chemical communication occurs. The ECS occupies approximately 20% of the brain volume and appears to be an interconnected meandrous network resembling a spider web with an estimated average width of a few tens of nanometers.[1] The ECS is a reservoir of ions essential for proper electrical activity of neurons. Various signaling molecules (e.g., neuro- and gliotransmitters, neuromodulators), hormones, and nutrients from the blood stream, as well as metabolites that need to be cleared out from brain, can diffuse through the brain tissue over short (between cells) or long (between regions) distances.[2] Within the ECS, there is a meshwork of polymeric molecules and diverse small proteins, called the extracellular matrix (ECM), which is known to regulate synaptic transmission and plasticity, network functions, and animal behavior.[3,4] Using an array of macroscopic biophysical and classical microscopy approaches in brain slices and *in vivo*, it has been shown that the volume and composition of the brain ECS vary during development, aging, sleep-wake cycles, as well as in neurodegenerative and neuropsychiatric diseases.[1,5] Only recently has the fine structure of the ECS been unveiled in live brain tissue using super-resolution microscopy techniques based on single-molecule imaging and stimulated emission depletion (STED) microscopy.[6,7] These studies provided the nanoscale ECS maps in live native tissue, demonstrating that, within a given region, the brain ECS is larger than predicted and highly heterogeneous in its width (ranging from tens to several hundreds of nanometers) and local viscosity. Results from electron microscopy based on cryo-fixation, which preserves ECS integrity, unlike chemical fixation, is consistent with these dimensions and heterogeneity.[8]

Our understanding of ECS fine organization in the living brain is thus still in its infancy. Since it is widely accepted that ECS geometry and composition ought to greatly influence the navigation of molecules between brain cells, decrypting ECS nanoscale organization in key brain structures remains an important open question. This applies in particular to finely structured brain areas, such as hippocampus, in which cellular elements are differentially mapped depending on their spatial localization. The hippocampus is a complex circuit made of functionally and molecularly distinct areas and layers that play central roles in learning and memory processes as well as cognitive tasks.[9] Glutamatergic synapses in distinct hippocampal areas display different molecular composition, plasticity range, glutamate spillover, as well as physiological interplay with neighboring cells such as astrocytes.[10,11] Furthermore, major hippocampal areas, i.e., dentate gyrus (DG), CA3, and CA1, exhibit differential vulnerability to various brain insults.[12–14] Therefore, the hypothesis that cell communication specificity within a given hippocampal area/layer is shaped by ECS characteristics and its impact on signaling molecule navigation is gaining experimental support. Although nano- and macroscopic examinations suggested that

This is an open access article under the CC BY license (http://creativecommons.org/licenses/by/4.0/).

hippocampal ECS volume fraction and tortuosity differ between DG, CA3, and CA1 areas,[1,15] major discrepancies between studies left this question unsolved.[15–20] Here, we used a combination of single-nanoparticle tracking and super-resolution microscopy approaches to measure the dimensions of hippocampal ECS and how biomolecules navigate within native ECS mazes.

RESULTS

QD-based exploration of the hippocampal extracellular space

Single-nanoparticle tracking provides a powerful way to define, at the nanoscale, some of the ECS characteristics in living brain tissue.[6,21–23] Quantum dots (QDs) are small nanocrystals that are brighter and more stable against photobleaching than most standard fluorescent dyes.[24] QDs have been successfully used to decipher several biological processes in the brain, such as membrane neurotransmitter receptor and transporter trafficking.[25–28] Here, rat cultured hippocampal slices were exposed to biocompatible QDs at 7 days *in vitro* (DIV) (QDs emitting at 655 nm) that were detected at the single-molecule level using a spinning disk confocal microscope (Figure 1A; see STAR Methods). We previously measured the hydrodynamic radius of these QDs at 22 nm,[29] a size compatible with the exploration of brain ECS (range from 30 nm up to 1–2 μm[6–8]). The QDs were detected and tracked at a depth range of 30–50 μm from the slice surface to avoid the first cell layers (which usually exhibit damage as consequence of the slicing process) and keep a good signal to noise ratio (Figure S1). Additionally, the integrity of the brain tissue was assessed after image processing and reconstruction of QD trajectories (Figure S2). The confined area of QD-based trajectories was then determined from the mean-square displacement (MSD) versus time lag curves. To ascertain that single QDs were mainly exploring the ECS, we performed several lines of experiments. First, QD-based trajectories were analyzed in hippocampal slices exposed to hyaluronidase for several hours in order to efficiently dissolve the ECM (Figure S3). The rationale was that, if QDs are navigating within the ECS, the ECM digestion would strongly increase QD-based trajectories, whereas, if QDs are mainly intracellular, this manipulation will have no major impact. Consistent with a previous study,[6] hyaluronidase left-shifted the MSD curves of the QD-based trajectories, indicating that the QDs were less confined following ECM digestion (Figures 1B and 1C). The MSD plateau, representing the confined area, was three times increased after 4 h of exposure to hyaluronidase (Figure 1C). These data already indicate that the vast majority of detected QDs are within the ECS. Second, because QDs can be efficiently endocytosed in active neurons,[30] we tested whether a well-established blocker of endocytosis, i.e., the dynamin inhibitor Dyngo4a,[31] alters QD-based trajectories. If a fraction of QDs are endocytosed by brain cells, this should be reflected in altered trajectory characteristics. We report that Dyngo4a failed to alter the MSD curves and confined areas (Figures 1C and 1D), as well as diffusion coefficients. Third, we manipulated neuronal activity and looked for potential effects on QD-based trajectories since it has been shown that synaptic and network activity affect brain ECS.[1] Blocking action potentials with tetrodotoxin (TTX) (15 min) strongly decreased the explored area of QD-based trajectories (Figures 1D and 1E), indicating an inactivity-dependent narrowing of the hippocampal ECS. Note that QDs themselves do not affect spontaneous synaptic activity of hippocampal neurons (Figure S4). Fourth, it has been suggested that the ECS volume fraction decreases during brain development.[32] We compared the MSD curves at 7 and 14 DIV, as a proxy of *in vitro* development, and report a consistent decreased confinement area in the *cornu ammonis* (CA) areas over time (Figure S5). Finally, the MSD curves and diffusion coefficients of either QD525 or QD655 nm were undistinguishable (Figure S6). Collectively, these experiments ascertain that virtually all detected QDs diffused within the ECS in our recording imaging session, irrespective of the hippocampal areas and QD types.

To establish ECS nanoscale maps in various hippocampal areas (i.e., DG, CA3, and CA1) and layers (e.g., *stratum oriens*, *stratum pyramidale*, and *stratum radiatum*) we tracked the diffusion of QDs (Figure 2A). We observed a striking difference in the *s. radiatum* between CA1 and CA3 areas, whereas the other layers were comparable (Figure 2B). Namely, QD-based trajectories were significantly less confined and more mobile in the *s. radiatum* of CA1 compared with CA3 (Figure 2B). At the whole-hippocampus level, the MSD and diffusion coefficient also greatly vary across areas and layers (Figure 2C). For instance, in the CA1 area, QD-based trajectories were gradually less confined and faster from *s. oriens* to *s. radiatum*. Furthermore, DG, CA3, and CA1 areas have overall distinct ECS characteristics (Figure 2C). To further ascertain these differences, we ran a spatial analysis that is based on the shape of the area boundaries explored by the detected QDs and not by the reconstructed trajectories (see STAR Methods). The dimensions of the QD-based areas were significantly reduced in the *s. radiatum* of CA3 compared with CA1 (Figures 2D and S7), consistent with the higher confinement in the CA3 area. Moreover, we tested whether QD-based ECS objects had similar orientation in CA3 and CA1 *s. radiatum* (Figures S7C and S7D). We found that most QD-based objects in the CA3 area do not have preferred orientation, whereas the majority of QD-based objects in the CA1 area have a preferred orientation (Figure S7). Collectively, these nanoscale maps of the ECS highlight its large heterogeneity across hippocampal areas and layers, with a noticeable difference between *s. radiatum* of CA1 and CA3 areas. To gain insight on the origin of the differences between sub-areas, we exposed hippocampal slices to 4 h of hyaluronidase treatment (Figure 2E). The confinement area was measured (see STAR Methods) and compared with the same parameter from an untreated slice from the same culture series. The distribution of ECS confinement values upon enzymatic ECM digestion in the *s. radiatum* of CA1 experienced only a mild shift toward greater explored spaces (Figure 2E, left graph). On the contrary, the explored area in the *s. radiatum* of CA3 significantly increased as a consequence of hyaluronidase treatment compared with untreated controls (Figure 2E, left graph), indicating that the ECM structure in CA3 has a greater influence on molecular diffusion than in CA1. Such a shift is further illustrated by the percentage change in ECS confinement distribution (see STAR Methods), which is a fair indicator of the percentage variation of the area explored

CellPress
OPEN ACCESS

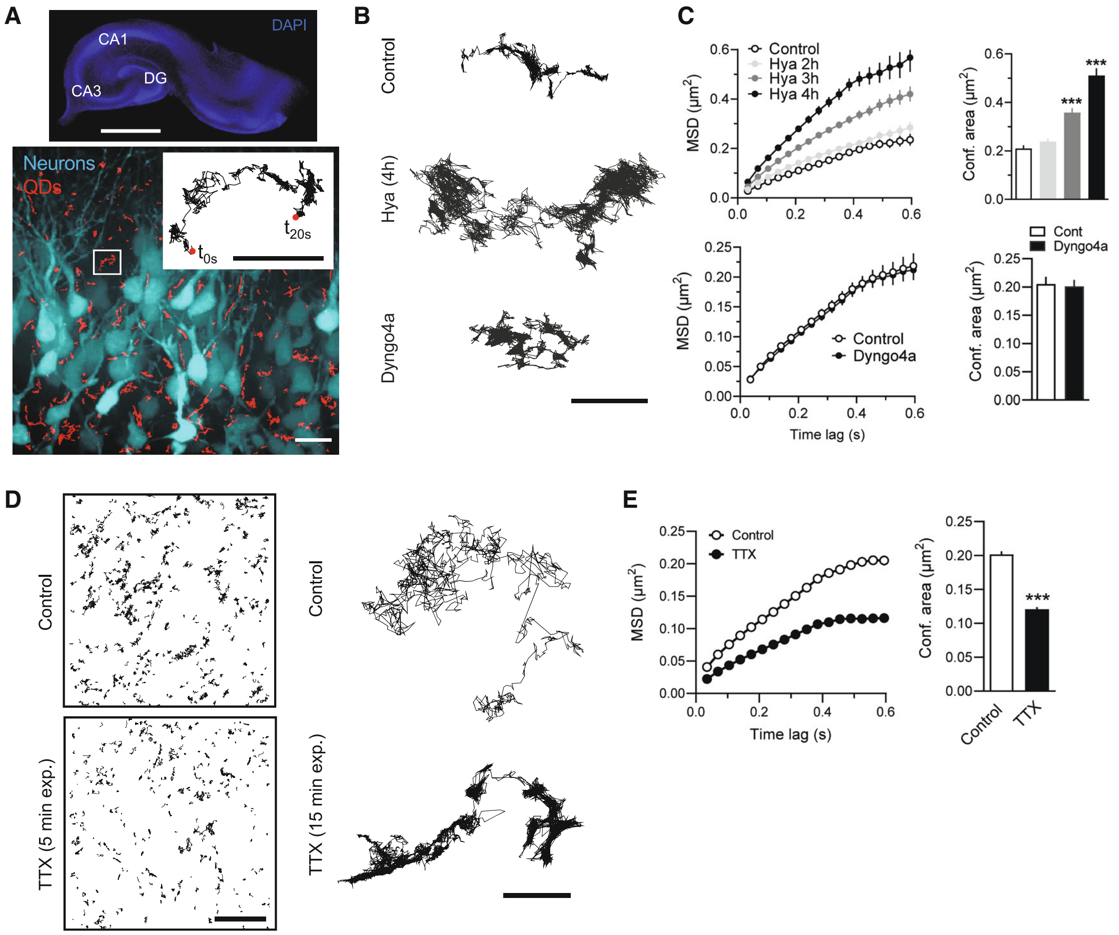

Figure 1. SPT-based exploration of the hippocampal extracellular space at the nanoscale level

(A) Summary of the experimental design to track QDs in organotypic brain slices. Upper panel: epifluorescence image of an hippocampal slice labeled with DAPI (scale bar, 500 μm). The slices were incubated with functionalized QDs. Lower panels: merged image showing reconstructed QDs trajectories (red traces) over an epifluorescence micrography of transduced neurons from DG area (scale bar, 15 μm); right: enlarged image (white inset) of a reconstructed QD trajectory (scale bar, 5 μm).

(B) Comparison of QD trajectories observed in control (upper panel), hyaluronidase (middle panel), and Dyngo-4a (lower panel) conditions obtained from organotypic slices (scale bar, 5 μm) (control = 1,817 trajectories, 24 slices; hyaluronidase 2 h = 1,817 trajectories, 20 slices; hyaluronidase 3 h = 1,812 trajectories, 19 slices; hyaluronidase 4 h = 877 trajectories, 11 slices).

(C) MSD versus time plots and confinement areas represented by the mean ± SEM values for hyaluronidase (upper panels) and Dyngo-4a (lower panels) experiments. Confinement areas were calculated based on their corresponding MSD curves. The number of trajectories are as follows: control = 2,140 trajectories (35 slices), Dyngo-4a = 1,860 trajectories (40 slices). *$p < 0.05$; ***$p < 0.001$, Kruskal-Wallis test.

(D) Comparison of representative QD trajectories (right panels, scale bar, 2 μm) and field of views of CA3 area showing QD trajectories (left panels, scale bar, 15 μm) observed in control and TTX-treated brain slices. The wide-field QD trajectories (left) correspond to 5 min after treatment for both control and TTX. Right, two representative QD trajectories after 5-min exposure to buffer or TTX.

(E) MSD versus time plot and confinement areas represented by the mean ± SEM values for control and TTX conditions after 15-min exposure to treatment. The number of trajectories are as follows: control = 10,954 trajectories (78 slices), TTX = 14,579 trajectories (59 slices). ***$p < 0.001$, Kruskal-Wallis test.

under hyaluronidase treatment compared with control (Figure 2E, right). Thus, the QD-based diffusional heterogeneity of ECS between CA3 and CA1 *s. radiatum* is almost completely absent after ECM digestion.

SUSHI-based exploration of the CA1–CA3 areas

The above QD-based measurements of the ECS are, by definition, reflecting the environment explored by the QDs. Because the size, surface chemistry, and other characteristics of the QD may alter or restrict their access to parts of the ECS, we used a complementary approach to measure the ECS dimensions in CA1–CA3 areas of the hippocampus. Super-resolution shadow imaging (SUSHI) is based on fluorescent labeling of the interstitial fluid and STED imaging of the brain tissue.[7] The extracellular labeling strategy using small fluorescent dyes greatly alleviates the distribution issue by filling up the whole ECS. As previously described,[7] the ECS of the mouse hippocampus can be directly visualized with this approach (Figure 3A). We thus measured and compared ECS dimensions of the *s. radiatum* in CA1 and CA3 areas (Figures 3B and S8) in organotypic mouse brain slices,

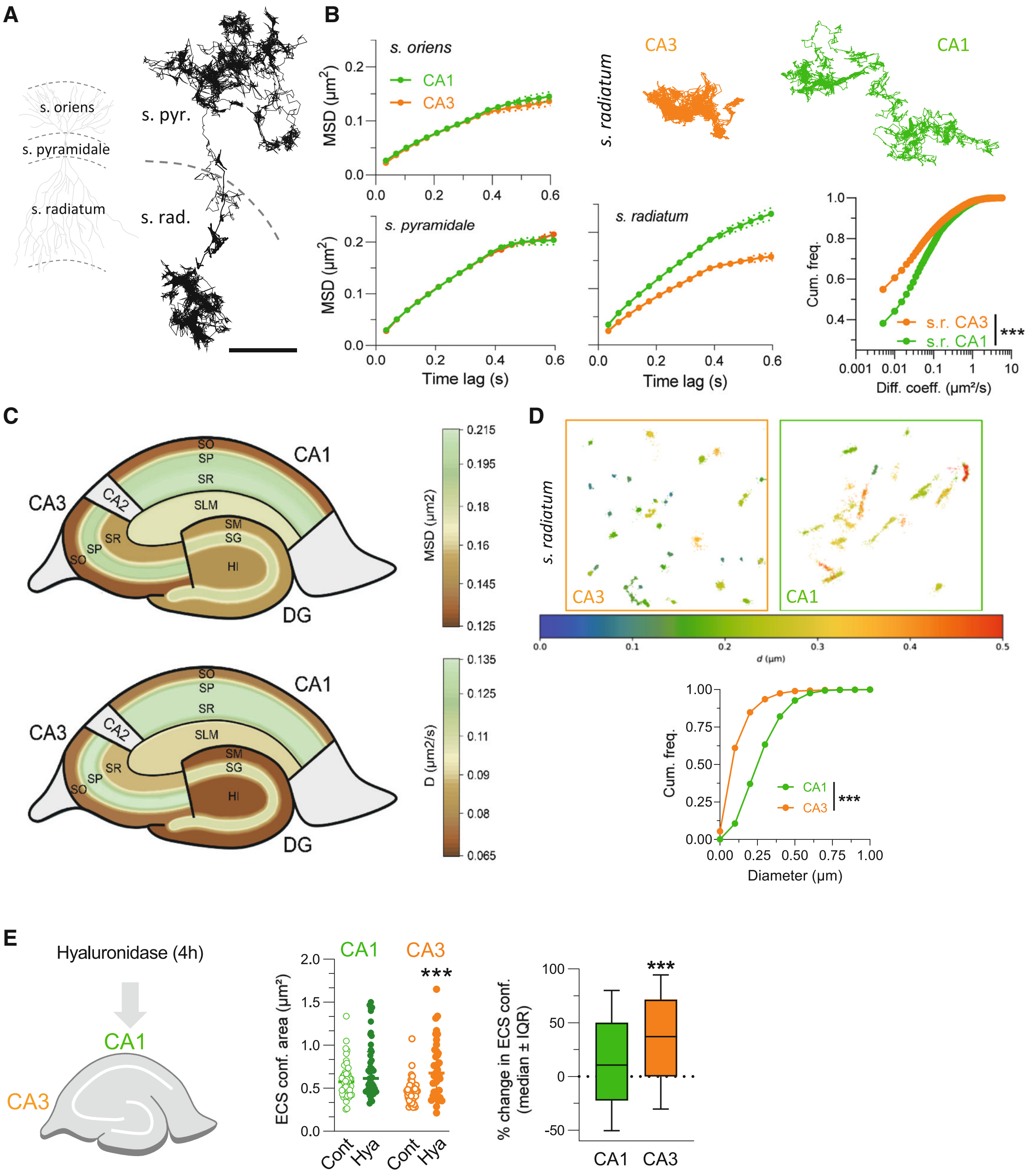

Figure 2. Dynamics heterogeneity of the hippocampal extracellular space at the nanoscale level

(A) Schematic of a pyramidal neuron (left) and representative example of a QD exploring *s. radiatum* and *s. pyramidale* (right). This long trajectory corresponds to a single QD tracked for almost 9,000 frames, uncovering a transition between *s. pyramidale* and *s. radiatum*. Dotted lines represent transitions between strata. Scale bar, 5 μm.

(B) MSD versus time plots for *s. oriens* and *s. pyramidale* in CA3 and CA1 areas (left panels). Representative QDs trajectories, MSD versus time and D. coefficient plots for *s. radiatum* in CA3 and CA1 areas (middle and right panels) (*s. oriens* CA3 = 3,946 trajectories, 44 slices; *s. pyramidale* CA3 = 20,255 trajectories, 315 slices; *s. radiatum* CA3 = 13,214 trajectories, 178 slices; *s. oriens* CA1 = 3,982 trajectories, 40 slices; *s. pyramidale* CA1 = 8,921 trajectories, 127 slices; *s. radiatum* CA1 = 13,214 trajectories, 107 slices; *stratum lacunosum moleculare* CA1 = 5,799 trajectories, 80 slices; *s. moleculare* DG = 6,310 trajectories, 97 slices; *stratum granulosum* DG = 4,500 trajectories, 81 slices; *hilus* DG = 4,911 trajectories, 55 slices. ***$p < 0.001$, Kolmogorov-Smirnov test). Scale bar, 5 μm.

(C) Topographic maps for MSD (upper panel) and diffusion coefficient (lower panel) values obtained in different hippocampal areas.

(D) Representative insets showing diameter variations for *s. radiatum* in CA3 and CA1 areas (upper panels) and cumulative fractions plot of measured diameters using a QD-based cloud points analysis (lower panel).

(E) Schematic representation of specific ECM components enzymatic digestion (left). Distributions of ECS confinement area values obtained from each recording are shown for untreated (Cont; CA1, n = 45 and CA3, n = 42, from 11 slices) and hyaluronidase-treated (Hya; CA1, n = 47 and CA3, n = 41, from 12 slices) slices (left graph). Right: the percentage variation in ECS confinement (medians ± IQR) between the two conditions (Cont vs. Hya) in the CA1 and CA3 *s. radiatum* areas (***$p < 0.001$, Mann-Whitney test).

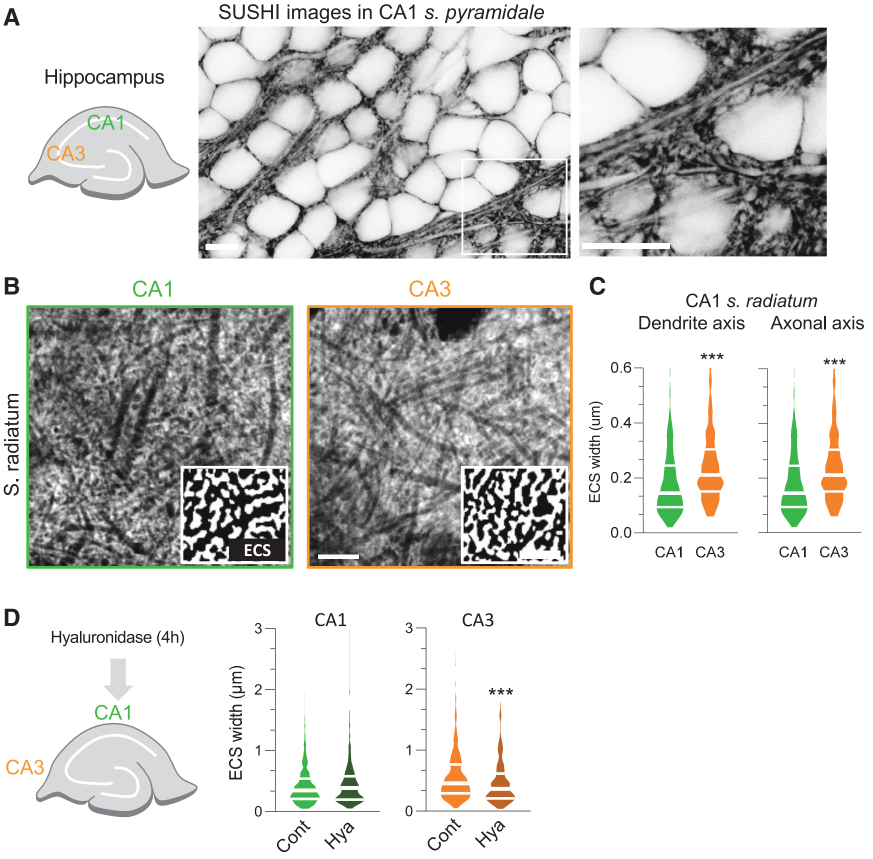

Figure 3. SUSHI-based exploration of the CA1-CA3 areas

(A) Left panel: Schematic representation of a DIV7 mouse hippocampal slice depicting CA3 and CA1 areas. Middle panel: a representative SUSHI image showing the cell layer of hippocampal CA1 area in *s. pyramidale*. Right panel: higher magnification of SUSHI image showing cell bodies and neuropil (white) and the ECS (black). Scale bars, 10 μm.

(B) SUSHI images of *s. radiatum* in CA1 (left) and CA3 (right) areas. Boxes in bottom right show segmented images representing enlarged sections of the ECS (white) and cellular elements (black). Scale bars, 10 μm, 5 μm, respectively.

(C) ECS widths plots for *s. radiatum* in CA1 and CA3 areas for the dendritic axis (i.e., parallel to the dendrites; n_{CA1} = 357 widths, n_{CA3} = 298 widths) and axonal axis (i.e., parallel to Schaffer collateral axons; n_{CA1} = 418 widths, n_{CA3} = 358 widths). $^{*}p < 0.05$, $^{***}p < 0.001$, Mann-Whitney test.

(D) ECS widths plots for *s. radiatum* in CA1 (n_{cont} = 866 widths, n_{Hya} = 351 widths) and CA3 (n_{cont} = 884 widths, n_{Hya} = 264 widths) areas (dendrite axis) after 4 h of hyaluronidase treatment and under control conditions. $^{*}p < 0.05$; $^{***}p < 0.001$, Mann-Whitney test.

where the technique had originally been developed. Schematically, the width of the super-resolved extracellular fluorescence between cell elements was measured in *s. radiatum* in both dendritic (from cell body to apical dendrite) and axonal (from CA3 to CA1 fields, perpendicular to the apical dendritic axis) axes. Consistent with the QD-based data, the ECS widths between CA1 and CA3 areas were significantly different for both axes (Figure 3C). As the QD-based ECS data were obtained in rat hippocampus, we also performed SUSHI measurements in organotypic rat brain slices, which were consistent with the results from mouse slices (Figure S9). In contrast to the QD measurements, the ECS in the *s. radiatum* is, however, wider in the CA3 than in the CA1 area (Figure 3C). These complementary approaches indicate that the anatomical width of the ECS and the ability of nanoparticles to diffuse therein can in fact diverge. Because the difference in QD-based ECS dimensions between CA3 and CA1 *s. radiatum* was virtually gone after digestion of the ECM, we performed a similar series of experiments using SUSHI imaging. Slices were exposed to hyaluronidase for 4 h (Figures 3D and S10), and the ECS width was measured and compared with the same parameter sampled in untreated companion slices. The distribution of ECS width upon enzymatic ECM digestion in CA1 *s. radiatum* was not altered (Figure 3D). On the contrary, ECS width in CA3 *s. radiatum* significantly decreased as a consequence of hyaluronidase treatment compared with untreated controls (Figures 3D and S10). In ECM digested conditions, the difference of ECS width between CA1 and CA3 *s. radiatum* disappeared (CA1, ECS width 0.45 ± 0.02 μm, n = 351; CA3, 0.47 ± 0.02 μm, n = 254). Thus, SUSHI-based CA3 and CA1 *s. radiatum* ECS nanoscale dimensions equalize following digestion of the ECM.

Molecular diffusion and distribution in the CA1 and CA3 ECS

The heterogeneity of the hippocampal ECS, such as in the *s. radiatum* of CA1 and CA3 areas, suggests that extracellular molecule dynamics and distribution may differ between areas. To directly test this possibility at the single-nanoparticle level, we exposed hippocampal slices to a solution of QDs and measured over time their surface density in CA1 and CA3 areas. The prediction is that ECS characteristics influence QD distribution. Consistently, the QD density was significantly higher in the CA1 *s. radiatum* compared with the CA3 area (Figures 4A and 4B), whereas the densities between CA1 and CA3 *s. oriens* and *pyramidale* were not statistically different (Figure 4B). These observations indicate that the higher dynamics of QDs in CA1 *s. radiatum* (compared with CA3) are associated with a higher dispersion. The distribution of nanoparticles within the hippocampal tissue is thus not homogeneous and likely depends on ECS characteristics: higher dynamics, although in a smaller ECS compartment, favor the accumulation of nanoparticles.

Whether this relationship between ECS nanoparticle dynamics and accumulation applies to biomolecules is of prime interest to further understand the biophysics of molecule diffusion in brain tissue. To directly address this question, we tracked one of the most abundant proteins of the brain fluid, the immunoglobulin

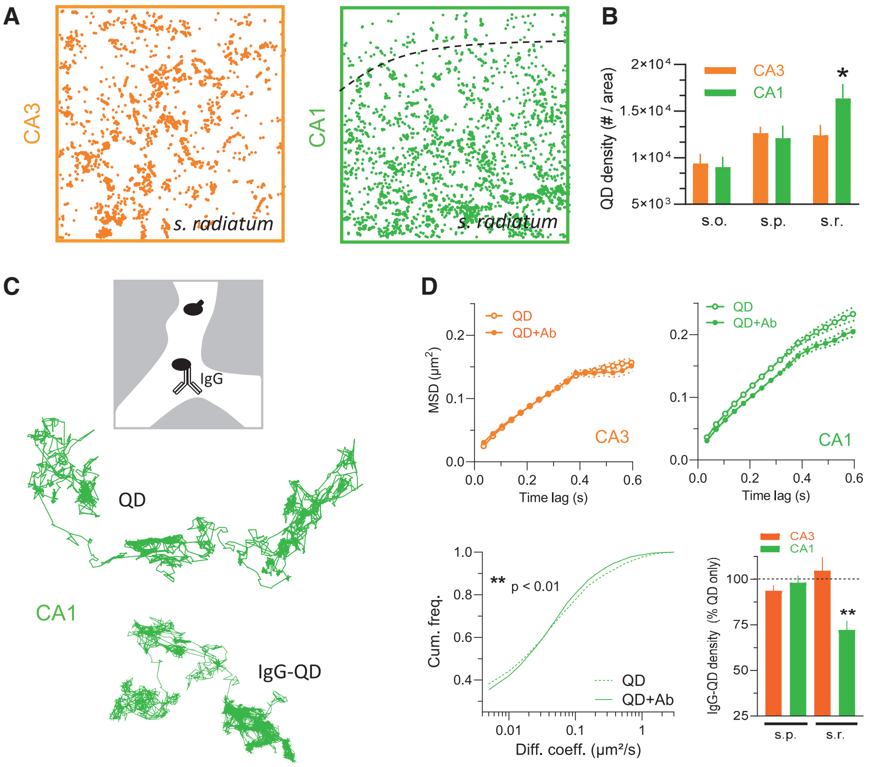

Figure 4. Distribution of biological molecules in the CA1 and CA3 areas

(A) Fields of view showing QDs detected (i.e., nanoparticles deposition) in *s. radiatum* of CA3 (left panel) and CA1 (right panel) areas (scale bar, 15 μm).

(B) Bar graphs of QD detected in each layer (s.p = *s. oriens*, s.p. = *s. pyramidale*, and s.r. = *s. radiatum*) of CA1 and CA3 areas represented by the mean ± SEM values.

(C) Schematic representation showing distribution of QD alone or IgG-QD complexes on the hippocampal ECS (upper panel). Comparison of representative trajectories observed for control QD alone (QD) and QD-IgG complexes (IgG-QD) experiments on the *s. pyramidale* of the CA1 area (scale bar, 5 μm).

(D) MSD versus time plots comparing QDs alone and QDs coupled to a neutral IgG (i.e., an IgG that has no target in the brain) experiment's impact on the *s. pyramidale* of the CA3 and CA1 areas (upper panels). Coefficient plot comparing QDs alone and QDs coupled to a neutral IgG experiment's impact on the *s. pyramidale* of the CA1 area and (lower panel, left). **$p < 0.01$, Kolmogorov-Smirnov test. Lower right panel, IgG-QD densities in CA1 and CA3 areas. For each hippocampal area, IgG-QD density is expressed as a percentage of the IgG-QD versus QD only. For *s. radiatum* CA1, the IgG-QD density is reduced over 30% compared with the QDs not bound to IgG density (*s. pyramidale* CA3 = 11,406 trajectories, 113 slices; *s. radiatum* CA3 = 5,384 trajectories, 63 slices; *s. pyramidale* CA1 = 8,636 trajectories, 78 slices), *s. radiatum* CA1 = 6,234 trajectories, 56 slices; **$p < 0.01$, Kruskal-Wallis test).

type G (IgG), in the *s. radiatum* of CA1 and CA3 areas. We used a neutral IgG (no target in the brain, IgG directed against GFP) that was coupled to QDs and tracked over time to define its ECS dynamics and local density (Figure 4C). Based on the above ECS diffusional and anatomical heterogeneity, one could make two predictions. First, the ECS behavior of IgGs is differentially affected in CA1 and CA3 areas. Indeed, our data show that the ECS behavior of IgG-QDs was different than that of the QDs only in the CA1 but not in the CA3 area (Figures 4C and 4D). In the CA1 area, IgG-QDs were more confined with an overall reduced dynamics, whereas there was no variation in the CA3 area. The second prediction is based on the above assumption that a higher dynamic favors the accumulation of nanoparticle, so reduced ECS dynamics of IgG should also reduce their accumulation. We thus compared the IgG densities in CA1 and CA3 areas focusing on *s. pyramidale* and *radiatum*. As predicted, the IgG-QD density was specifically and significantly reduced in the CA1 *s. radiatum* (Figure 4D). Altogether, these data indicate that the ECS characteristics of the CA1 (not CA3) *s. radiatum* strongly affect the dynamics and accumulation of extracellular molecules.

DISCUSSION

Decrypting the role of the ECS and its components in brain physiology remains an important frontier in neuroscience. Although the ECS occupies around a fifth of brain volume and all molecules involved in brain cell communication necessarily navigate across ECS, it remains one of the least understood major compartments of the brain. This was mostly due to lack of appropriate techniques to visualize live ECS nanoanatomy and study its biophysical properties. Here, we took advantage of single-QD tracking and super-resolution microscopy approaches to measure the nano-dimensions of hippocampal ECS in the live hippocampal formation. The ECS dimensions strongly differ between areas of the rodent hippocampus, with a striking divergence between CA1 and CA3 areas in the *s. radiatum*. Moreover, we tested the ECS navigation of single molecules in these defined ECS nanoscale mazes. Consistent with the distinct ECS characteristics between areas, we report that the ECS dynamics and distribution of antibodies differ across these areas. Collectively, we provide a nanoscale map of the hippocampal network and show the role of such heterogeneity for molecule dynamics.

The brain ECS is a critical, albeit overlooked, compartment of the brain. Progress has been driven by waves of technological innovation. These advances have, almost exclusively, been focused on various intracellular processes, leaving our comprehension of the extracellular environment at a rudimentary level. Most of our knowledge about the spatial organization of the ECS is inferred from pioneering macroscopic biophysical

investigations, such as real-time iontophoresis, integrative optical imaging, diffusion-weighted magnetic resonance imaging, and electron microscopy.[33] These technologies operate on complementary scales, where the biophysical approaches can extract average structural parameters, such as ECS volume fraction and tortuosity, while electron microscopy can reveal tissue ultrastructure in fixed samples. However, using these approaches to define the volume fraction and tortuosity of the ECS in CA1 and CA3 area, these studies provided conflicting results.[16–20] For instance, ECS volume fraction and tortuosity were reported to be smaller in CA1 than CA3,[16,20] whereas other studies reported that both volume fraction and tortuosity were higher in CA1 than CA3.[34,35] Discrepancies are possibly related to different methodological approaches, biological preparations, and/or developmental stages.

Based on our development of tracking of single-nanoparticle and super-resolution SUSHI imaging, we are able to access the nanoscale dimension of the hippocampal ECS in living slices. Indeed, single-QD tracking provides information on how a given nanometric object moves in the ECS and thus the dimensions explored by such an object, whereas the SUSHI approach provides information on the ECS dimensions in a nanoparticle-independent manner. Note that the ECS dimensions provided by our single-QD tracking are comparable with the ones described with single-wall carbon nanotubes (SWCNTs) (QD based, ~260 nm width, 20–1,500 nm range; SWCNTs, ~180 nm width, 50–700 nm range[6]). The fact that we obtained different results strengthens the view that these complementary approaches need to be used in tandem to better grasp the components that shape brain ECS. Globally, our nanoscale mapping confirms that the hippocampus is a highly heterogeneous structure also at the level of the ECS. Our data support a model in which the ECS volume is larger in CA3 than CA1 areas. However, the observation that nanoparticles were more confined in the CA3 suggests that (1) the ECS spatial organization is more complex in the CA3 area (e.g., presence of dead ends or concave 3D elements related to CA3 curvature), and/or (2) the ECM in the CA3 is molecularly different and/or denser, and thus more prone to affect molecule dynamics. Although more investigations are needed to shed light on these possibilities, the observation that the ECM digestion in the CA3 strongly "de-confines" nanoparticles and alters ECS widths suggests an influential role of the matrix. To address that, a systematic analysis of local differences in ECM composition will provide more insights into regional ECS heterogeneities and reconcile the results obtained by single-particle tracking (SPT) and SUSHI.

Limitation of the study

This study provides a nanoscale map of the hippocampal ECS, demonstrating high local heterogeneity. Our data were obtained from hippocampal organotypic slices, an *in vitro* preparation that preserves the main architecture of the hippocampus but exhibit differences with its *in vivo* counterpart. For instance, the flattening of the hippocampal slices over weeks might overall distort the ECS architecture. Furthermore, the *in vitro* preparation used in our study did not address the potential impact of sex differences of the hippocampal ECS. As juvenile rats (2–3 weeks after birth) show sexual dimorphism in the hippocampal perineuronal net formation,[36] it is possible that male and female hippocampal ECS vary. Characterizing the hippocampal ECS in freely moving male and female rodents is an unmet challenge that will surely complement and shed additional light on the nanoscale ECS map of the hippocampus.

STAR★METHODS

Detailed methods are provided in the online version of this paper and include the following:

- KEY RESOURCES TABLE
- RESOURCE AVAILABILITY
  - Lead contact
  - Materials availability
  - Data and code availability
- EXPERIMENTAL MODEL AND SUBJECT DETAILS
  - Organotypic brain slices
- METHOD DETAILS
  - Nanoparticles preparation
  - Fluorescence labelling and imaging
  - Image acquisition and single particle tracking (SPT) analysis
  - Shape analysis of QD-covered areas
  - Super-resolution shadow imaging (SUSHI)
  - Dyngo-4a treatment
  - Hyaluronidase treatment
  - Tetrotodoxin (TTX) assay
  - Immunohistochemistry of organotypic brain slices with aggrecan
  - Viral infection of organotypic slices
  - Calcium imaging
- QUANTIFICATION AND STATISTICAL ANALYSIS

SUPPLEMENTAL INFORMATION

Supplemental information can be found online at https://doi.org/10.1016/j.celrep.2023.112478.

ACKNOWLEDGMENTS

We thank the Bordeaux Imaging Center, a service unit of the CNRS-INSERM and Bordeaux University, member of the national infrastructure France BioImaging (ANR-10-INBS-04-01). We would like to thank Corey Butler and Adel Kechkar for their contribution to PALMTracer; the Cell Biology Facility, especially Delphine Bouchet, Constance Manso, and Emeline Verdier, for cell biology activity management; and lab members for constructive discussions, especially Hélène Gréa and Frédéric Villéga. This work was supported by the postdoctoral Marie-Curie European fellowship (NanoPSYCH, #845542 to D.G.), Centre National de la Recherche Scientifique, Agence Nationale de la Recherche (ANR-14-OHRI-0001-01, ANR-15-CE16-0004-03), Fondation pour la Recherche Médicale, Human Frontier Science Program (RGP0019 to L.G. and RGP0041/2019 to U.V.N.), European Research Council Synergy grant (ENSEMBLE,#951294 to L.G., U.V.N., and L.C.), Conseil Régional d'Aquitaine, Labex Bordeaux BRAIN, post-doctoral fellowship of the Fondation ARC pour la Recherche sur le Cancer (to A.L.), and a PhD fellowship from Bordeaux Neurocampus Graduate Program (to A.I.).

AUTHOR CONTRIBUTIONS

D.G., A.I., and I.C. generated resources and performed and analyzed experiments. A.I. and V.N. performed and analyzed SUSHI imaging experiments.

A.L. and L.C. performed the single-nanoparticle mapping analysis. J.-B.S. developed the analytical tool for single-nanoparticle tracking and dynamics. D.G. and L.G. conceived and supervised the project, designed the experiments, and wrote the manuscript with input from co-authors.

DECLARATION OF INTERESTS

The authors declare no competing interests.

Received: May 20, 2022
Revised: February 17, 2023
Accepted: April 20, 2023
Published: May 5, 2023

REFERENCES

1. Syková, E., and Nicholson, C. (2008). Diffusion in brain extracellular space. Physiol. Rev. *88*, 1277–1340.
2. Nicholson, C., Chen, K.C., Hrabětová, S., and Tao, L. (2000). Diffusion of molecules in brain extracellular space: theory and experiment. Prog. Brain Res. *125*, 129–154.
3. Dityatev, A., Schachner, M., and Sonderegger, P. (2010). The dual role of the extracellular matrix in synaptic plasticity and homeostasis. Nat. Rev. Neurosci. *11*, 735–746.
4. Fawcett, J.W., Oohashi, T., and Pizzorusso, T. (2019). The roles of perineuronal nets and the perinodal extracellular matrix in neuronal function. Nat. Rev. Neurosci. *20*, 451–465.
5. Rasmussen, M.K., Mestre, H., and Nedergaard, M. (2022). Fluid transport in the brain. Physiol. Rev. *102*, 1025–1151.
6. Godin, A.G., Varela, J.A., Gao, Z., Danné, N., Dupuis, J.P., Lounis, B., Groc, L., and Cognet, L. (2017). Single-nanotube tracking reveals the nanoscale organization of the extracellular space in the live brain. Nat. Nanotechnol. *12*, 238–243.
7. Tonnesen, J., Inavalli, V., and Nagerl, U.V. (2018). Super-resolution imaging of the extracellular space in living brain tissue. Cell *172*, 1108–1121.
8. Korogod, N., Petersen, C.C.H., and Knott, G.W. (2015). Ultrastructural analysis of adult mouse neocortex comparing aldehyde perfusion with cryo fixation. Elife *4*, e05793.
9. Goode, T.D., Tanaka, K.Z., Sahay, A., and McHugh, T.J. (2020). An integrated index: engrams, place cells, and hippocampal memory. Neuron *107*, 805–820.
10. Allen, N.J., and Lyons, D.A. (2018). Glia as architects of central nervous system formation and function. Science *362*, 181–185.
11. Sherwood, M.W., Oliet, S.H.R., and Panatier, A. (2021). NMDARs, coincidence detectors of astrocytic and neuronal activities. Int. J. Mol. Sci. *22*, 7258.
12. Bartsch, T., Döhring, J., Reuter, S., Finke, C., Rohr, A., Brauer, H., Deuschl, G., and Jansen, O. (2015). Selective neuronal vulnerability of human hippocampal CA1 neurons: lesion evolution, temporal course, and pattern of hippocampal damage in diffusion-weighted MR imaging. J. Cerebr. Blood Flow Metabol. *35*, 1836–1845.
13. Roussarie, J.P., Yao, V., Rodriguez-Rodriguez, P., Oughtred, R., Rust, J., Plautz, Z., Kasturia, S., Albornoz, C., Wang, W., Schmidt, E.F., et al. (2020). Selective neuronal vulnerability in alzheimer's disease: a network-based analysis. Neuron *107*, 821–835.e12.
14. Alkadhi, K.A. (2019). Cellular and molecular differences between area CA1 and the dentate gyrus of the Hippocampus. Mol. Neurobiol. *56*, 6566–6580.
15. Zheng, K., Jensen, T.P., Savtchenko, L.P., Levitt, J.A., Suhling, K., and Rusakov, D.A. (2017). Nanoscale diffusion in the synaptic cleft and beyond measured with time-resolved fluorescence anisotropy imaging. Sci. Rep. *7*, 42022.
16. McBain, C.J., Traynelis, S.F., and Dingledine, R. (1990). Regional variation of extracellular space in the hippocampus. Science *249*, 674–677.
17. Hrabetová, S. (2005). Extracellular diffusion is fast and isotropic in the stratum radiatum of hippocampal CA1 region in rat brain slices. Hippocampus *15*, 441–450.
18. Saghyan, A., Lewis, D.P., Hrabe, J., and Hrabetova, S. (2012). Extracellular diffusion in laminar brain structures exemplified by hippocampus. J. Neurosci. Methods *205*, 110–118.
19. Arranz, A.M., Perkins, K.L., Irie, F., Lewis, D.P., Hrabe, J., Xiao, F., Itano, N., Kimata, K., Hrabetova, S., and Yamaguchi, Y. (2014). Hyaluronan deficiency due to Has3 knock-out causes altered neuronal activity and seizures via reduction in brain extracellular space. J. Neurosci. *34*, 6164–6176.
20. Pérez-Pinzón, M.A., Tao, L., and Nicholson, C. (1995). Extracellular potassium, volume fraction, and tortuosity in rat hippocampal CA1, CA3, and cortical slices during ischemia. J. Neurophysiol. *74*, 565–573.
21. Thorne, R.G., and Nicholson, C. (2006). In vivo diffusion analysis with quantum dots and dextrans predicts the width of brain extracellular space. Proc. Natl. Acad. Sci. USA *103*, 5567–5572.
22. Varela, J.A., Dupuis, J.P., Etchepare, L., Espana, A., Cognet, L., and Groc, L. (2016). Targeting neurotransmitter receptors with nanoparticles in vivo allows single-molecule tracking in acute brain slices. Nat. Commun. *7*, 10947.
23. Wang, Z.G., Wang, L., Lamb, D.C., Chen, H.J., Hu, Y., Wang, H.P., Pang, D.W., and Liu, S.L. (2021). Real-time dissecting the dynamics of drug transportation in the live brain. Nano Lett. *21*, 642–650.
24. Wegner, K.D., and Hildebrandt, N. (2015). Quantum dots: bright and versatile in vitro and in vivo fluorescence imaging biosensors. Chem. Soc. Rev. *44*, 4792–4834.
25. Dahan, M., Lévi, S., Luccardini, C., Rostaing, P., Riveau, B., and Triller, A. (2003). Diffusion dynamics of glycine receptors revealed by single-quantum dot tracking. Science *302*, 442–445.
26. Groc, L., and Choquet, D. (2020). Linking glutamate receptor movements and synapse function. Science *368*, eaay4631.
27. Groc, L., Heine, M., Cognet, L., Brickley, K., Stephenson, F.A., Lounis, B., and Choquet, D. (2004). Differential activity-dependent regulation of the lateral mobilities of AMPA and NMDA receptors. Nat. Neurosci. *7*, 695–696.
28. Murphy-Royal, C., Dupuis, J.P., Varela, J.A., Panatier, A., Pinson, B., Baufreton, J., Groc, L., and Oliet, S.H.R. (2015). Surface diffusion of astrocytic glutamate transporters shapes synaptic transmission. Nat. Neurosci. *18*, 219–226.
29. Rosendale, M., Flores, J., Paviolo, C., Pagano, P., Daniel, J., Ferreira, J., Verlhac, J.B., Groc, L., Cognet, L., and Blanchard-Desce, M. (2021). A bottom-up approach to red-emitting molecular-based nanoparticles with natural stealth properties and their use for single-particle tracking deep in brain tissue. Adv. Mater. *33*, e2006644.
30. Park, H., Li, Y., and Tsien, R.W. (2012). Influence of synaptic vesicle position on release probability and exocytotic fusion mode. Science *335*, 1362–1366.
31. Neef, J., Jung, S., Wong, A.B., Reuter, K., Pangrsic, T., Chakrabarti, R., Kügler, S., Lenz, C., Nouvian, R., Boumil, R.M., et al. (2014). Modes and regulation of endocytic membrane retrieval in mouse auditory hair cells. J. Neurosci. *34*, 705–716.
32. Larsen, B.R., Stoica, A., and MacAulay, N. (2019). Developmental maturation of activity-induced K(+) and pH transients and the associated extracellular space dynamics in the rat hippocampus. J. Physiol. *597*, 583–597.
33. Hrabetova, S., Cognet, L., Rusakov, D.A., and Nägerl, U.V. (2018). Unveiling the extracellular space of the brain: from super-resolved microstructure to in vivo function. J. Neurosci. *38*, 9355–9363.
34. Mazel, T., Simonová, Z., and Syková, E. (1998). Diffusion heterogeneity and anisotropy in rat hippocampus. Neuroreport *9*, 1299–1304.

CellPress
OPEN ACCESS

35. Syková, E., Vorísek, I., Mazel, T., Antonova, T., and Schachner, M. (2005). Reduced extracellular space in the brain of tenascin-R- and HNK-1-sulphotransferase deficient mice. Eur. J. Neurosci. *22*, 1873–1880.

36. Griffiths, B.B., Madden, A.M.K., Edwards, K.A., Zup, S.L., and Stary, C.M. (2019). Age-dependent sexual dimorphism in hippocampal cornu ammonis-1 perineuronal net expression in rats. Brain Behav. *9*, e01265.

37. Levet, F., Tønnesen, J., Nägerl, U.V., and Sibarita, J.B. (2020). SpineJ: a software tool for quantitative analysis of nanoscale spine morphology. Methods *174*, 49–55.

STAR★METHODS

KEY RESOURCES TABLE

REAGENT or RESOURCE	SOURCE	IDENTIFIER
Antibodies		
Donkey anti-mouse Alexa Fluor 568	Thermo Scientific	Cat #A-10037
Donkey anti-rabbit QD 525	Thermo Scientific	Cat #Q22074
Goat anti-rabbit Alexa Fluor 488	Thermo Scientific	Cat #A-11070
Goat anti-rabbit QD 655	Thermo Scientific	Cat #Q11422MP
Mouse anti-tenascin-R	Synaptic Systems	Cat #217 011
Rabbit anti-aggrecan	Merck Millipore	Cat #AB1031
Rabbit anti-GFP	Thermo Scientific	Cat# A6455
Biological samples		
Postnatal day 5 hippocampal organotypic slices (details described in Experimental model and Subject detail below)	This paper	N/A
Chemicals, peptides, and recombinant proteins		
Tetrodotoxin (TTX) citrate	Bio-Techne	Cat #1069/1
Dyngo 4a	ABCAM	Cat #ab120689
Dimethyl sulfoxide	Sigma-Aldrich	Cat #276855
Bovine serum albumin	Sigma-Aldrich	Cat #A3059
Hyaluronidase (from Streptomyces hyalurolyticus)	Sigma-Aldrich	Cat #H1136
Fluoromount-G mounting medium containing DAPI	Thermo Scientific	Cat #00-4959-52
Calcein	Dojindo Laboratories	Cat #C001
Experimental models: Organisms/strains		
Sprague-Dawley rats	Janvier labs	N/A
Recombinant DNA		
Lentivirus encoding YFP under Synapsin promotor	Provided by C. Mulle	LU 110-2
Software and algorithms		
Prism 8 software	GraphPad	https://www.graphpad.com/scientific-software/prism/
MetaMorph software (7.8.9.0)	Molecular Devices	https://www.moleculardevices.com/systems/metamorph-research-imaging/metamorph-microscopy-automation-and-image-analysis-software
ImageJ software	National Institutes of Health	http://imagej.nih.gov/ij
PALM Tracer package	Custom	https://neuro-intramuros.u-bordeaux.fr/displayresearchprojects/70/11
NPD.view2 software	Hamamatsu	https://www.hamamatsu.com/eu/en/product/type/U12388-01/index.html
Custom shape analysis routines implemented in Python and in C++ (available upon request)		https://scientific-python.org/

RESOURCE AVAILABILITY

Lead contact

Further information and requests for resources and reagents should be directed to the lead contact, Laurent Groc (laurent.groc@u-bordeaux.fr).

Materials availability

This study did not generate any unique reagents.

Data and code availability

This paper does not report original code. Any additional information required to reanalyze the data reported in this paper is available from the lead contact upon request.

EXPERIMENTAL MODEL AND SUBJECT DETAILS

Postnatal 5-days-old Sprague-Dawley rat pups (Janvier/Charles River, France) were used for this work, both male and female, treated according to the guidelines of the University of Bordeaux/CNRS Animal Care and Use Committee.

Organotypic brain slices

Hippocampal organotypic slices were obtained from postnatal 5-days-old Sprague-Dawley rat pups, both male and female (Janvier/Charles River, France). Dissection and culture preparation were performed under laminar flow hood in sterile conditions. The animals were quickly decapitated, and their brains placed on cold sterile dissection medium containing (in mM): 0.5 CaCl2, 2.5 KCl, 2 MgCl2, 0.66 KH2PO4, 0.85 Na2HPO4-12H2O, 0.28 MgSO4-7H2O, 50 NaCl, 2.7 NaHCO3, 25 glucose, 175 sucrose, 2 HEPES (all products from Sigma unless specified). The hippocampi were dissected and then sliced on a McIlwain tissue chopper to generate coronal brain slices of 350 mm thickness. After 20 min at 4°C, the resulting slices were evaluated to choose only those presenting both intact Cornu Ammonis (CA) and Dentate Gyrus (DG) areas. The selected slices were transferred on small pieces (~5 × 5 mm) of sterilized hydrophilic polytetrafluoroethylene (PTFE) membrane (FHLC04700, Merck Millipore) which were set on top of Millicell cell culture inserts (Millipore, 0.4 mm; ∅ 30 mm). Growing brain slices over PTFE membranes facilitated the subsequent recovery of the slices without having to detach them from the insert membrane and therefore minimizing the impact on the ECS architecture. The inserts were placed in 6-wells multiwell filled with preheated culture medium composed of 50% Basal medium eagle (BME), 25% Hank's Balanced Salt Solution (HBSS), 25% Horse Serum Gibco, 11.2 mmol/L glucose and 20 mM GlutaMAX (all products from GIBCO unless specified) and cultured for up to 7 days at 35°C / 5% CO2. Culture medium was completely replaced (1 mL/well) the day after plating of slices and then every 3 days until performing experiments. Since the ECS architecture shows variation over time on *ex vivo* preparations, the slices were used at 7 days after plating to perform all the experiments. At that time, the slices are recovered from the trauma caused by dissection procedure.

METHOD DETAILS

Nanoparticles preparation

Free QDs: QD655 goat F(ab')2 anti-rabbit IgG (Q11422MP, Invitrogen) or QD525 donkey anti-rabbit IgG (Q22074, Invitrogen) were diluted in warm ACSF at a working concentration of 1:5000. Nonspecific binding was blocked by adding bovine serum albumin (BSA) 1% (A3059, Sigma) to the ACSF solution. This ACSF-BSA solution containing free QDs was applied to brain slices for 60 min at 35°C. The rationale of this procedure is to incubate the QDs with an excess of a given IgG (ratio 1:10) to ensure that all the QDs are coupled to IgGs and not free. Briefly, 0.5 μg of 655QDs were mixed in with 5 μg of a given IgG in 26 μL of PBS1X for 30 min at RT. Then, the QD-antibody complexes were diluted in warm ACSF-BSA 1% at a working concentration of 1:5000, and applied to brain slices for 75 min at 35°C. For those experiments performed with a neutral non-human IgG, a commercially available antibody targeting the green fluorescent protein (GFP, no brain target) was used (A6455, Invitrogen). This anti-GFP IgG were coupled to QD655 goat F(ab')2 anti-rabbit IgG (Q11422 MP, Invitrogen).

Fluorescence labelling and imaging

At day 7 after plating, organotypic slices were washed on HEPES-based ACSF containing (in mM): 130 NaCl, 2.5 KCl, 2.2 CaCl2, 1.5 MgCl2, 10 HEPES, and 10 D-glucose for 5 min to remove culture medium. Then, the slices were incubated with ACSF-BSA1% containing either free QDs or QDs-antibody complexes for a period ranging from 60 to 90 min according to the assay (see "Nanoparticles preparation"). Finally, the slices were washed for 5 min at 35°C and placed in a Ludin chamber for imaging. An electrophysiology harp was used to maintain the slices at the bottom of the chamber and evenly placed regarding the focal plane, which facilitated the image acquisition. The temperature of the chamber was kept at 35°C during all acquisition steps with a temperature controller device (Warner). The slices were imaged with an upright microscope Nikon Eclipse Ni-E equipped with a CSU-X1 confocal scanner unit (Yokogawa), and a motorized movable top plate (Scientifica). First, the slices were inspected at low magnification (10× objective) to evaluate their quality and discard those presenting damage. Then, the slices were imaged at a higher magnification (60× objective, 1.4 NA), QDs were detected using a mercury lamp and appropriate excitation/emission filters and recorded with an EMCCD camera (Evolve, Photometrics). The brain slices were used for a period of 20–25 min and then discarded.

Image acquisition and single particle tracking (SPT) analysis

Image acquisition was performed at a depth of 30–50 μm to avoid the first cell layers, which can present damage due to the slicing process. Images were acquired using Metamorph software (Molecular Devices) with an exposure time of 30 ms per image for up to 500 consecutive frames in the case of ECS dynamics analyses. To obtain representative trajectories as those showed in different figures, the acquisitions ranged from 4,000 to 8,000 consecutive frames. Image stacks were analyzed using PALMTracer software,

a dedicated program for single molecule localization and tracking based the combination of wavelet segmentation and Gaussian fitting for localization and simulated annealing algorithms for tracking. It allows both the localization of individual QD in all image plans and their tracking through successive images. The instantaneous diffusion coefficient, D, was calculated for each trajectory from linear fits of the first four points of the mean-square displacement (MSD) function versus time. Data were plotted using GraphPad Prism software. All trajectories shorter than 12 points were filtering out for accurate diffusion constant determination.

For the assays using QD525, numbers of measurements for each condition were: *s.pyramidale CA3* = 1897 trajectories (33 slices), *s.radiatum CA3* = 2749 trajectories (44 slices), *s.pyramidale CA1* = 1529 trajectories (20 slices), *s.radiatum CA1* = 1656 trajectories (16 slices).

For the ECS measurements at different developmental stages, data for each condition were: *s.pyramidale CA3* = 4558 trajectories (63 slices), *s.radiatum* CA3 = 3595 trajectories (43 slices), *s.pyramidale CA1* = 7208 trajectories (89 slices), *s.radiatum CA1* = 5573 trajectories (64 slices).

Comparisons between groups for confinement areas and nanoparticles densities were performed using Mann-Whitney test or Kruskal-Wallis followed by a Dunn's Multiple Comparison Test, as the variables are not following normal distributions. For direct comparison of distributions, Kolmogorov-Smirnov test was used.

Shape analysis of QD-covered areas

Visual examination of QD trajectories revealed that they covered either elongated (‘‘1D’’) areas or punctual (‘‘0D’’) areas with similar widths. To distinguish the two cases, covered areas were first fitted to ellipses; areas for which the ratio of long-to-short axis was less than sqrt(2) were deemed to be ‘‘0D’’ and assigned zero length. All other (‘‘1D’’) areas were fit using a polyline (a polygonal line), determined so as to minimize a combination of the mean squared distances of the individual localizations to the polyline and a penalty term based on the length of the polyline. For regularization purposes, the individual segments of the polyline were constrained to be of equal length. Finally, the total number of segments was selected by 2-fold cross-validation. This entire procedure was implemented using custom Python and C++ code. Polylines were then broken into individual segments, and the orientations of all the segments, weighted by their lengths, were put in histograms. This distribution of orientations was fitted either to a uniform angular distribution, to a circular normal distribution, or to a linear combination of two circular normal distributions with orthogonal centers. The best fit model was selected based on the Akaike information criterion.

Super-resolution shadow imaging (SUSHI)

STED microscopy

We used a home-built 3D-STED setup constructed around an inverted microscope body (DMI 6000 CS, Leica Microsystems), which was equipped with a TIRF oil objective (100X, 1.47 NA, HXC APO, Leica Microsystems) and placed inside an enclosed heating box (Cube and Box, Life Imaging Services) to keep the temperature stable at 32°C. A diode-laser (PDL 800-D, PicoQuant) was used to deliver pulsed excitation at 485 nm and another laser (Onefive Katana 06 HP, NKT Photonics) pulsed STED de-excitation at 594 nm. A spatial light modulator (Easy3D Module, Abberior Instruments) was used to generate a donut and/or bottle beam-shaped intensity distributions of the STED light to improve the spatial resolution in x, y and z. Image acquisition was controlled by the Imspector software (Abberior Instruments). The spatial light modulator was also used to correct spherical aberrations of the STED laser beam, using reflective (gold) nano-spheres to visualize the focal intensity distribution. The spatial resolution of the microscope was 175 nm (x-y) and 450 nm (z) in confocal mode and 60 nm (x-y) and 160 nm (z) in STED mode.

Extracellular labeling

For the extracellular staining, calcein (Dojindo Laboratories) was diluted in 1x ACSF (119 mM NaCl, 2.5 mM KCl, 1.3 mM $MgSO_4$, 26 mM $NaHCO_3$, 1mM NaH_2PO_4, 2.5 mM $CaCl_2$, 20 mM D-Glucose, 300 mOsm, pH 7.3) to a final concentration of 20 μM. A brain slice was mounted onto a glass coverslip inside imaging chamber and perfused with carbogenated calcein/ACSF solution for at least 10 min prior to imaging.

Hyaluronidase treatment

Rat organotypic hippocampal slices (DIV 10–14) were incubated with 20 mg/mL hyaluronidase enzyme (H-1136, Sigma) diluted in HEPES-based ACSF for 4h in the incubator. This was followed by extracellular labeling and STED imaging (same parameters as described above).

Acquisition parameters

STED single-plane images were acquired with the following acquisition parameters: 100 × 100 μm^2 field of view, 19.53 nm pixel size, 0.3 ms dwell time, 0.5 μW excitation laser power and 30 mW STED laser power (both measured after the objective).

ECS widths analysis and statistics

First, the images were binarized using the ImageJ plugin SpineJ[37] based on wavelet-filtering. Following, three parallel lines with the same interval were systematically drawn on the binarized images of *s.pyramidale* and six lines (divided in axonal and dendritic axes) for *s. radiatum*. The line profiles were then plotted using ImageJ software (NIH). Each peak width of the plot was then measured and considered as an individual ECS width (Figure S8A). For statistical analyses, GraphPad Prism 9 software was used. The data were tested for normal distribution and then Mann-Whitney test was performed to define the statistical significance. *S.radiatum* in CA1 and CA3 areas for the dendritic axis: $n_{\mathbf{CA1}}$ = 357 widths, $n_{\mathbf{CA3}}$ = 298 widths; axonal axis $n_{\mathbf{CA1}}$ = 418 widths, n_{CA3} = 358 widths. Hyaluronidase treatment: *s.radiatum* in CA1: $n_{\mathbf{cont}}$ = 866 widths, $n_{\mathbf{Hya}}$ = 351 widths; and CA3 $n_{\mathbf{cont}}$ = 884 widths, $n_{\mathbf{Hya}}$ = 264 widhts.

Dyngo-4a treatment

In order to abolish endocytosis before QD incubation, one-week old brain slices were pre-treated for 15 min with Dyngo-4a (ab120689, abcam) 20 μM diluted on ACSF. Since Dyngo-4a is a reversible dynamin inhibitor, to maintain the inhibitory effects the organotypic slices were incubated with ACSF-BSA 1% containing QD655 goat F(ab')2 anti-rabbit IgG (Q11422 MP, Invitrogen) at a dilution of 1:5000 and Dyngo-4a 5 μM for 60 min at 35°C. Then the slices were washed for 5 min and imaged in ACSF containing Dyngo-4a at a dilution of 5 μM. In the control condition the pre-treatment, incubation, washing, and imaging steps were performed using ACSF supplemented with equivalent amount of Dimethyl sulfoxide (DMSO, Sigma).

Hyaluronidase treatment

Alteration of the ECM was performed by using hyaluronidase, an enzyme that disrupt digest hyaluronic acid. One-week old brain slices were treated for a given period (2, 3 or 4–5 h) with Hyaluronidase (H-1136, Sigma) at a dilution of 20 mg/mL (70U/ml) on ACSF. In the control conditions the treatment step was performed incubating the slices on ACSF supplemented with equivalent amount of phosphate saline buffer 1X (PBS) for a given period (2, 3 or 4 h). The PBS- or hyaluronidase-treated slices were then washed for 15 min and processed for either perform single particle tracking experiments or to evaluate hyaluronic acid digestion. In the first case, the slices were incubated with QD655 goat F(ab')2 anti-rabbit IgG (Q11422 MP, Invitrogen) diluted at 1:5000 on ACSF-BSA 1% for 60 min at 35°C, then washed and imaged. In the latter case, samples were processed as explained on "Aggrecan staining" section. To provide an estimate of the ECM contribution to the differential confinement observed in some hippocampal subfields within the same *stratum* (*e.g. s. radiatum*), MSD values were isolated at the plateau (0.8–1 s). For each recording, the confinement area was calculated as the averaged MSD at the specified time lag in the plateau. In a subset of experiments, confinement was sampled in both untreated and hyaluronidase-treated (4 h) slices from the same culture series. This was instrumental for introducing a parameter defined as "percent change in ECS confinement" obtained through $\frac{ECS_{conf}\ treated - ECS_{conf}\ untreated}{\frac{ECS_{conf}\ treated + ECS_{conf}\ untreated}{2}} \times 100$.

By calculating this percent variation for all pairs of recordings in hyaluronidase *versus* untreated controls within the same hippocampal subfield, it was possible to obtain a distribution of the aforementioned parameter.

Tetrotodoxin (TTX) assay

Modification of the neuronal network activity was performed by using tetrodotoxin (TTX), a potent blocker of voltage gated sodium channels. Slices were incubated with ACSF-BSA 1% containing QD655 goat F(ab')2 anti-rabbit IgG (Q11422 MP, Invitrogen) at a dilution of 1:5000 for 60 min at 35°C, then washed for 5 min with ACSF and prepared to be imaged. Once a selected hippocampal area (CA3) was properly visualized at a given depth (~30 μm), the slice medium (ACSF alone) was replaced with warm ACSF containing TTX 1 μM and repeated acquisitions of 500 frames were performed each 90 s during 15 min. In the control condition the incubation, washing, hippocampal area selection, medium change and imaging steps were performed using ACSF-BSA 1% supplemented with equivalent amount of water.

Immunohistochemistry of organotypic brain slices with aggrecan

The staining was performed as follow: one-week old organotypic slices (control or hyaluronidase-treated) were washed for 30 min with Phosphate buffered saline (PBS) 1X, then fixed for 1 h with paraformaldehyde (PFA) 4% diluted in PBS 1X and finally rinsed 3 times (15 min each wash) with PBS 1X supplemented with Triton X-100 (TX) 0.01%. After removing this solution, the slices were incubated for 2 h with a blocking buffer containing Bovine serum albumin (BSA, Sigma, Cat. #A3059) 10% PBS 1X, TX 0.01%, and Glycine 0.3M. Then, the samples were incubated overnight at 4°C with anti-aggrecan rabbit antibody (Cat. #AB1031, Merck Millipore) at a dilution of 1:400. Thereafter, the slices were rinsed 3 times (15 min each wash) with PBS 1X - TX 0.01% and incubated for 2 h with a goat anti-rabbit Alexa Fluor 488 antibody (Cat. #A-11070, Thermo Fischer) at a concentration of 1:500 diluted in blocking buffer. Finally, the slices were washed 3 times with PBS 1X - TX 0.01% solution (15 min each rinse), mounted in superfrost ultra plus (Thermo Scientific Labs) slides and sealed using Fluoromount-G mounting medium containing DAPI (Cat. #00-4959-52). All the staining steps were performed at room temperature (RT). One day after sealing, the slides were imaged using a slides scanner (Hamamatsu NANOZOOMER 2.0HT Light) with a 20× objective (NA 0.75) and the resulting images were processed with dedicated viewing software (NPD.view2) and ImageJ.

Viral infection of organotypic slices

Organotypic slices at day 1 after plating were treated with viral particles encoding yellow fluorescent protein (YFP) as follow: small pieces (~3 × 3 mm) of sterilized PTFE membranes were soaked with ~0.5–1 μl of purified virus diluted in PBS and then added to the top (air interface) of each slice. After 2 days the membranes carrying the virus were removed and the culture of the infected slices continued for 5 days (as described previously) until being used for imaging experiments.

Calcium imaging

To explore synaptic activity upon acute QD exposure, neuronal cells were transfected by Ca3(PO4)2 method with a GCaMP6f construct (Plasmid #52924, Addgene), as fluorescent reporter for calcium dynamics. DNA (0.5 μg) of transgene per glass coverslip

were precipitated in a tube and added drop-wise on top of neuronal cultures at 8 DIV. Transfected cultures were allowed to express the exogenous gene for at least one week, after which neuronal activity was studied. Synaptic calcium dynamics were studied on hippocampal neurons grown for 14–18 DIV. Cultures were equilibrated for 5 min at room temperature (RT) in extracellular saline solution of composition (mM): 130 NaCl, 2.5 KCl, 2.2 CaCl2, 1.5 MgCl2, 10 HEPES, and 10 glucose (pH adjusted to 7.4 with NaOH; osmolarity ~300 mOsm). Samples were placed in a recording chamber mounted on an upright microscope (Eclipse Ni-E, Nikon) equipped with a confocal spinning disk unit (CSU-X1, Yokogawa). Genetically encoded Ca^{2+} reporter was excited at 488 nm with a 200-mW laser diode (L4Cc, Oxxius) at 4% power. Excitation light was separated from the emitted using a quad band polychroic mirror (405/488/561–568/635-647, Chroma); emitted light was further passed on a 525/50 nm single-band bandpass filter (BrightLine). Neurons were imaged with a 60× water dipping objective (CFI Apo NIR, 1 N.A.) and frames were continuously acquired each 150 ms using an Evolve 512 EMCCD camera (Teledyne Photometrics). The imaging system was controlled by an integrated acquisition software (NIS-Elements AR, Nikon). Extracellular saline was renewed prior recording, which was conducted at RT. Spontaneous neuronal activity was monitored for 8–10 min pre and post incubation (5 min) with 1 mL of commercial QDs (Invitrogen, ref. Q11422MP) at a final concentration of 150 pM. The selected amount corresponds to a higher concentration used during extracellular spaces (ECS) loading in organotypic hippocampal slices. As a proof of QD presence, the latter were tracked at 20 Hz for 20 s at the end of synaptic activity recordings. Briefly, QDs were excited at 405 nm with the same laser diode as above, emitted light was passed through the same polychroic mirror and further band-passed with a 600/52 nm filter (BrightLine). Acquired nanoparticles trajectories were finally reconstructed with PalmTracer software (MetaMorph, Molecular Devices). On average 7 regions of interest (ROI) per neuron were drawn around active spines (Figure S3). Synaptic events were count in each ROI to obtain median inter-event intervals (IEIs) and frequency values of spines. The activity of the latter was thus used to provide an estimate of single neurons behavior after nanoparticles exposure. From 6 different culture series, a total of 15 neurons from 15 different glass coverslips were studied. Time-lapse recordings were analyzed with ImageJ software (NIH), and the corresponding time series were studied with Clampfit software (pClamp suite, 11 version; Axon Instruments) in offline mode. Intracellular Ca^{2+} transients were expressed as fractional amplitude increase ($\Delta F/F0$, where F0 is the baseline fluorescence level and ΔF is the rise over baseline). Statistical analysis was performed using Prism 9 software (GraphPad): distribution normality was addressed with D'Agostino and Pearson omnibus normality test. Non-parametric tests were used in both non-normally distributed data and unequal variances among tested conditions. Accordingly, statistics between the two dependent variables were performed either with paired t test or Wilcoxon signed-ranks test.

QUANTIFICATION AND STATISTICAL ANALYSIS

The statistical analysis was performed with the help of GraphPad Prism 8 software (GraphPad Software, Inc). Statistical analyses are described in each corresponding section (see STAR Methods, above). Significance levels were defined as $^{*}p < 0.05$, $^{**}p < 0.01$, $^{***}p < 0.001$.

Cell Reports, Volume *42*

Supplemental information

Nanoscale and functional heterogeneity

# of the hippocampal extracellular space

Diego Grassi, Agata Idziak, Antony Lee, Ivo Calaresu, Jean-Baptiste Sibarita, Laurent Cognet, U. Valentin Nägerl, and Laurent Groc

Suppl. Figure 1

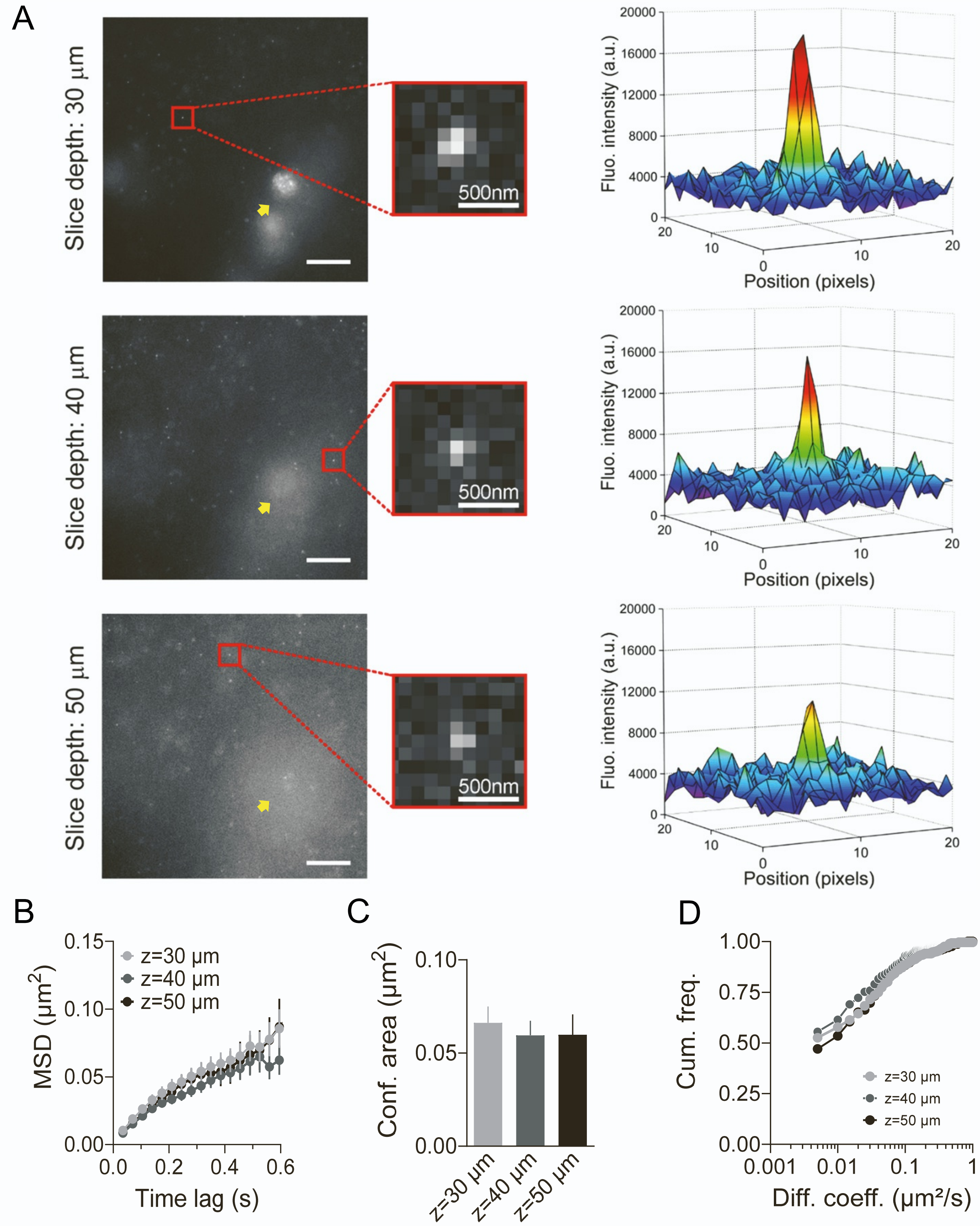

Figure S1. QD detection in hippocampal slice (Related to Figure 1)

(A) Left panles: QDs imaged at different depths (30, 40 and 50 µm) on a brain hippocampal slice (scale bars = 15 µm). Yellow arrows indicate the position of a cell body taken as reference. Red squares and enlarged insets show individual QDs visualized at different depths (scale bars, 500 nm). Right panels: heat map representation of fluorescence intensity of QDs indicated by red squares at different depths.

It should be noted that the QD tracking assays are usually performed at a depth of 30-50 µm since at depth below 50 µm the signal starts to exhibit significant degradation.

(B) MSD versus time plots for QD detections at different depths.

(C) Confinement areas represented by the mean ± SEM values for QD detections at different depths. Confinement areas were calculated based on their corresponding MSD curves.

(D) Diffusion coefficient cumulative distributions for QD recorded at different depths.

Suppl. Figure 2

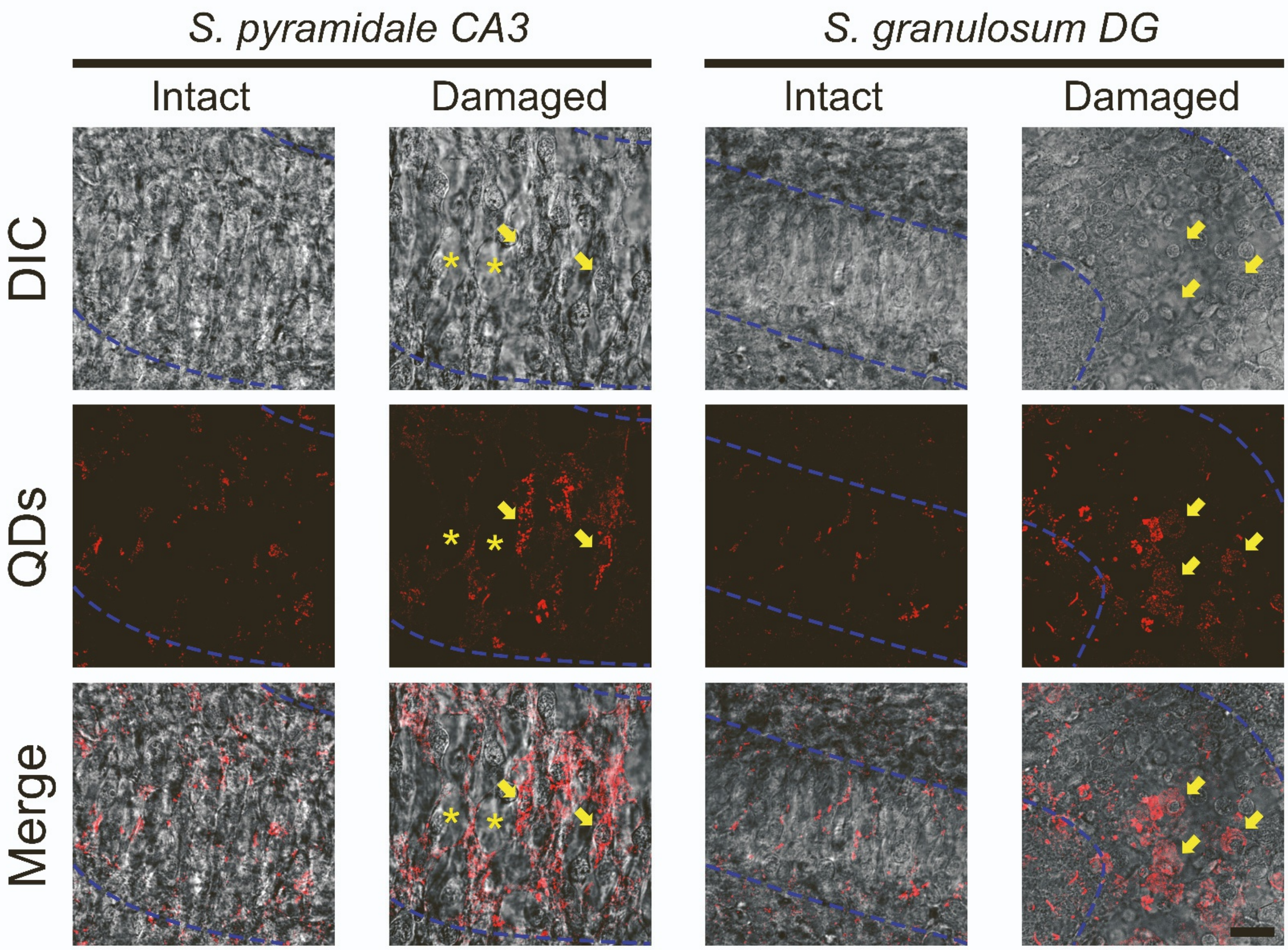

Figure S2. Quantum dot dispersion in intact or damaged brain tissue (Related to Figure 1)
Distribution of quantum dots in intact (first and third columns) or damaged (second and forth columns) tissue on DIV 7 organotypic brain slices (gray, DIC micrographies; red, quantum dots maximal projections of 500 frames). Cell layers of *s. pyramidale* CA3 and *s. granulosum* DG are delimited within blue dashed lines, yellow asterisks indicate cells exhibiting considerable swelling and yellow arrows mark dead cells with quantum dots invading the cytoplasm. Scale bar = 15 µm.

Suppl. Figure 3

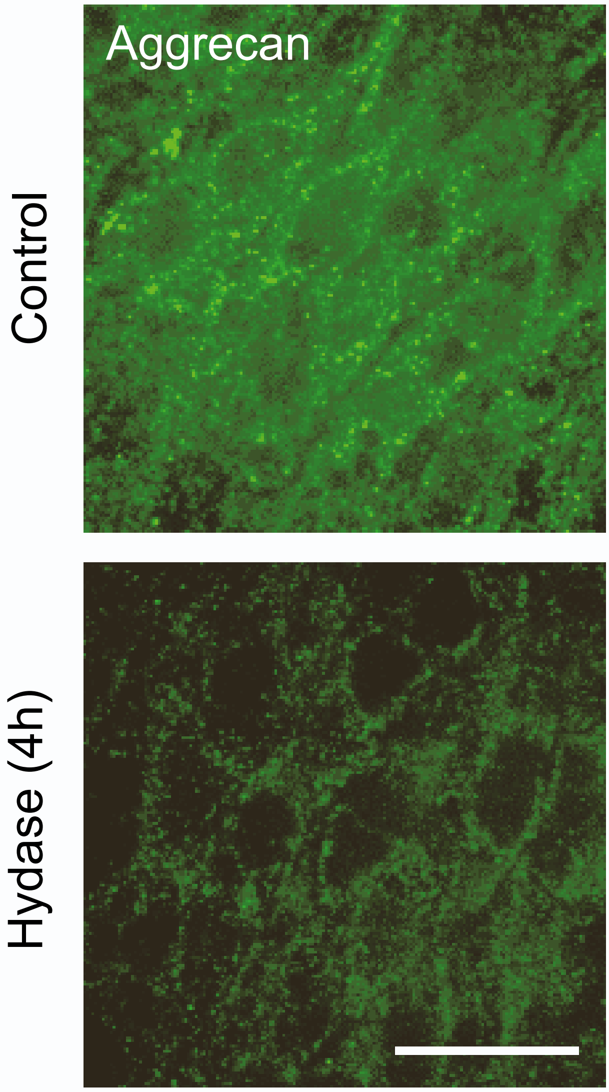

Figure S3. Effect of hyaluronidase on hippocampal ECM (Related to Figures 1 and 2)
Representative images of aggrecan immunostaining in control, untreated (upper panel) and hyaluronidase-treated (lower panel) CA3 hippocampal area (scale bar = 25 µm).

Suppl. Figure 4

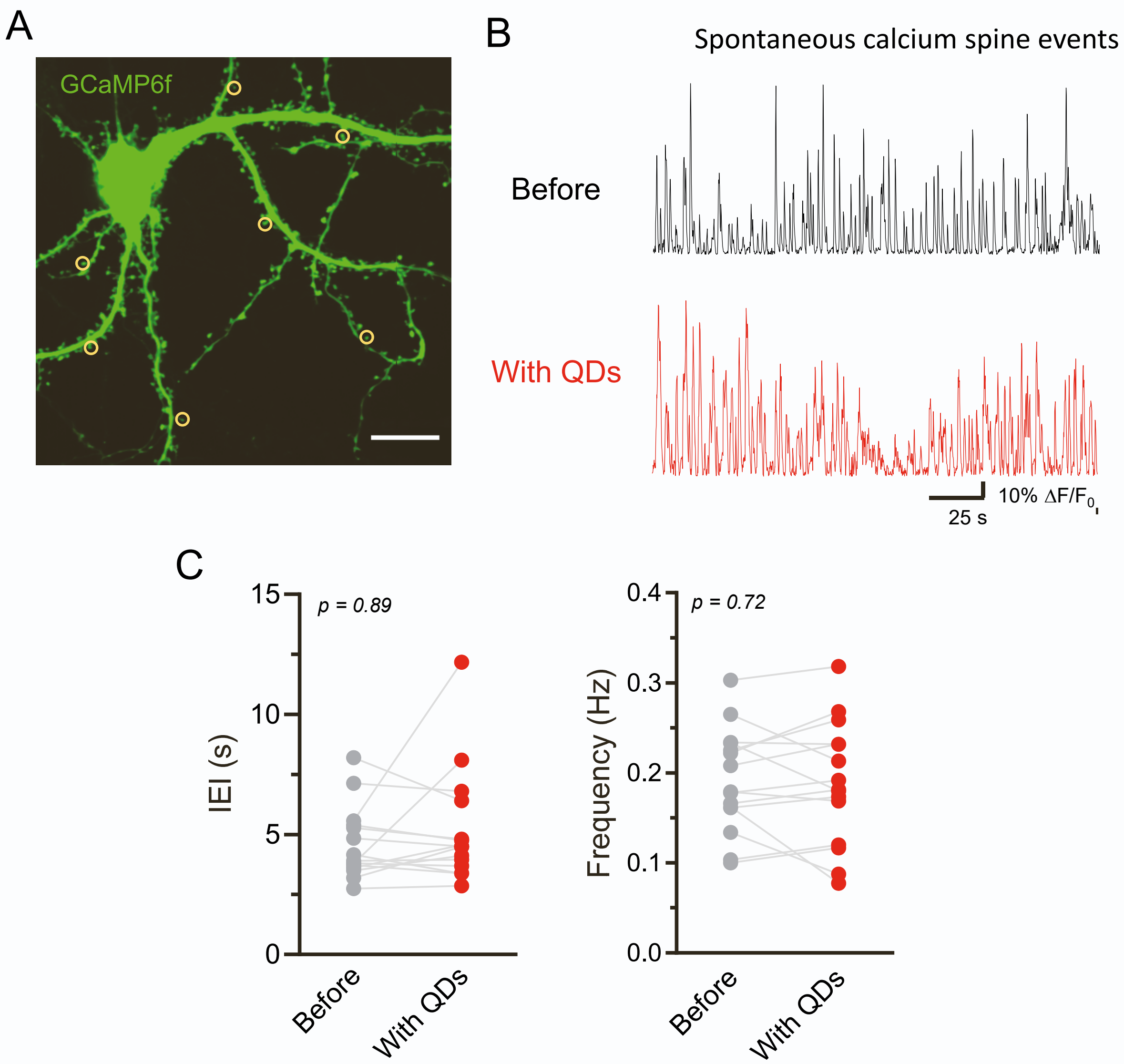

Figure S4. **Neuronal calcium dynamics recording from dendritic spines upon acute QD exposure (Related to Figure 1)**
(A) Snapshot of a representative field of view of a hippocampal neuron expressing GCaMP6f Ca2+ reporter is shown together with circular ROIs (yellow) around spines from which activity was monitored (Scale bar = 15 µm).
(B) Representative time series of Ca2+ transients recorded pre and post QD incubation are also provided (upper right). Calcium transients are expressed as fractional amplitude increase.
(C) Before and after plots illustrate averaged values of spines activity expressed as both inter event intervals (left) and frequency (right) for each recorded neuron. As denoted by p-values, under these experimental conditions no significative differences have been observed pre and post QD exposure.

Suppl. Figure 5

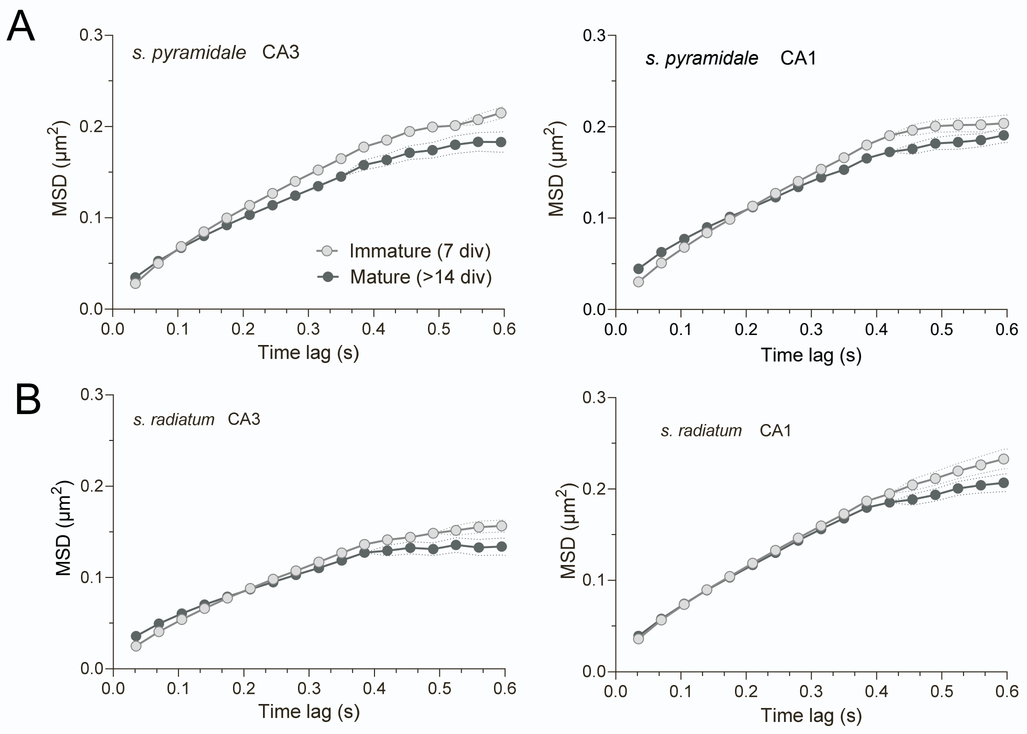

Figure S5. Comparison of QD-based ECS measurements at different developmental stages (Related to Figure 1)
(A) MSD versus time plots for *s. pyramidale* in CA3 (left panel) and CA1 (right panel) areas at 7 and >14 DIV.
(B) MSD versus time plots for *s. radiatum* in CA3 (left panel) and CA1 (right panel) areas at 7 and >14 DIV.

Suppl. Figure 6

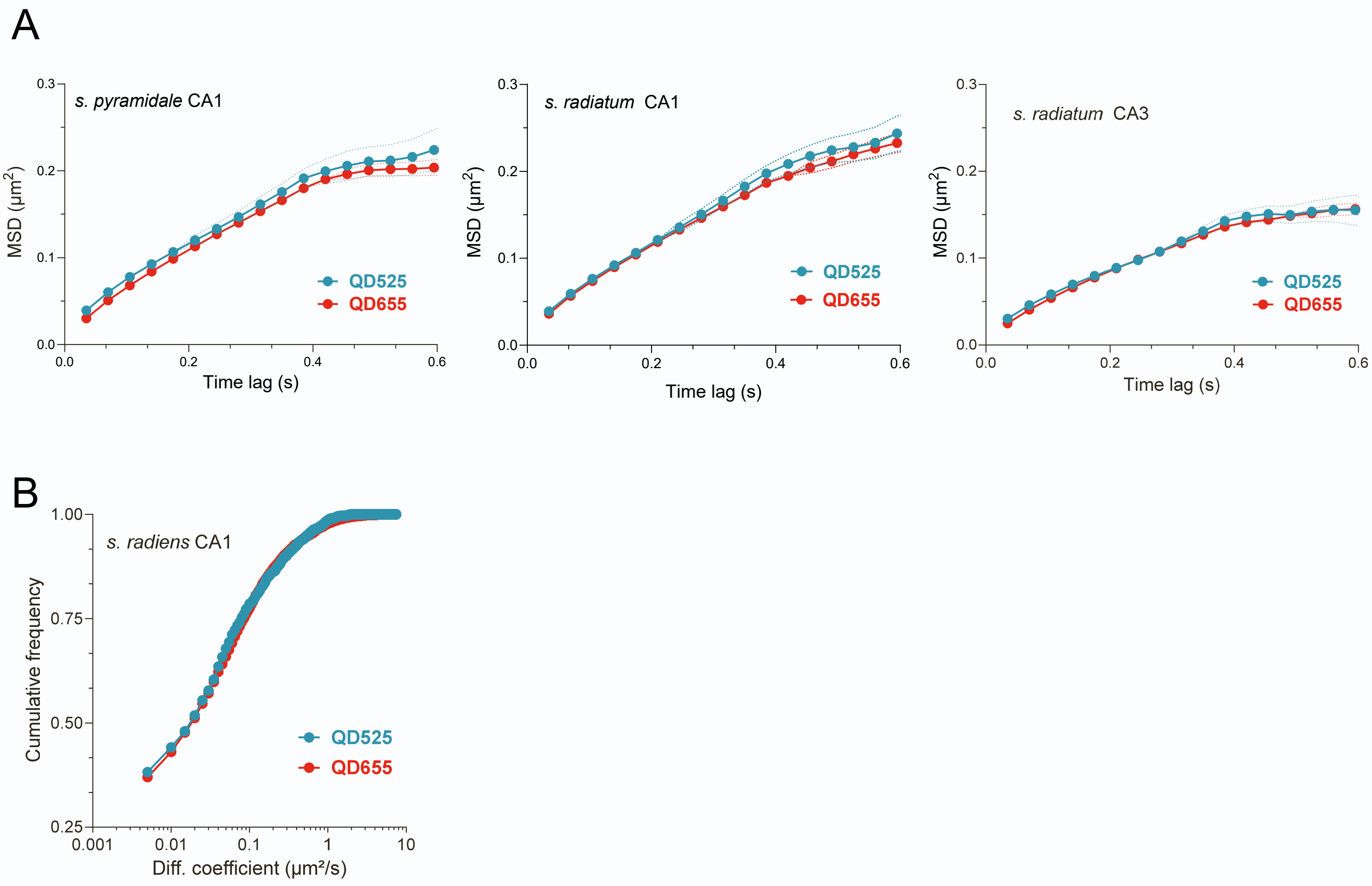

Figure S6. Comparison of QD-based ECS measurements from two different QD species (Related to Figure 1)
(A) MSD versus time plots of QD525 and QD655 for *s. pyramidale* in CA1 (left panel), *s. radiatum* in CA1 (middle panel) and *s. radiatum* in CA3 (right panel) areas.
(B) Diffusion coefficient cumulative distributions of QD525 and QD655 for s. *radiatum* in CA1 area.

Suppl. Figure 7

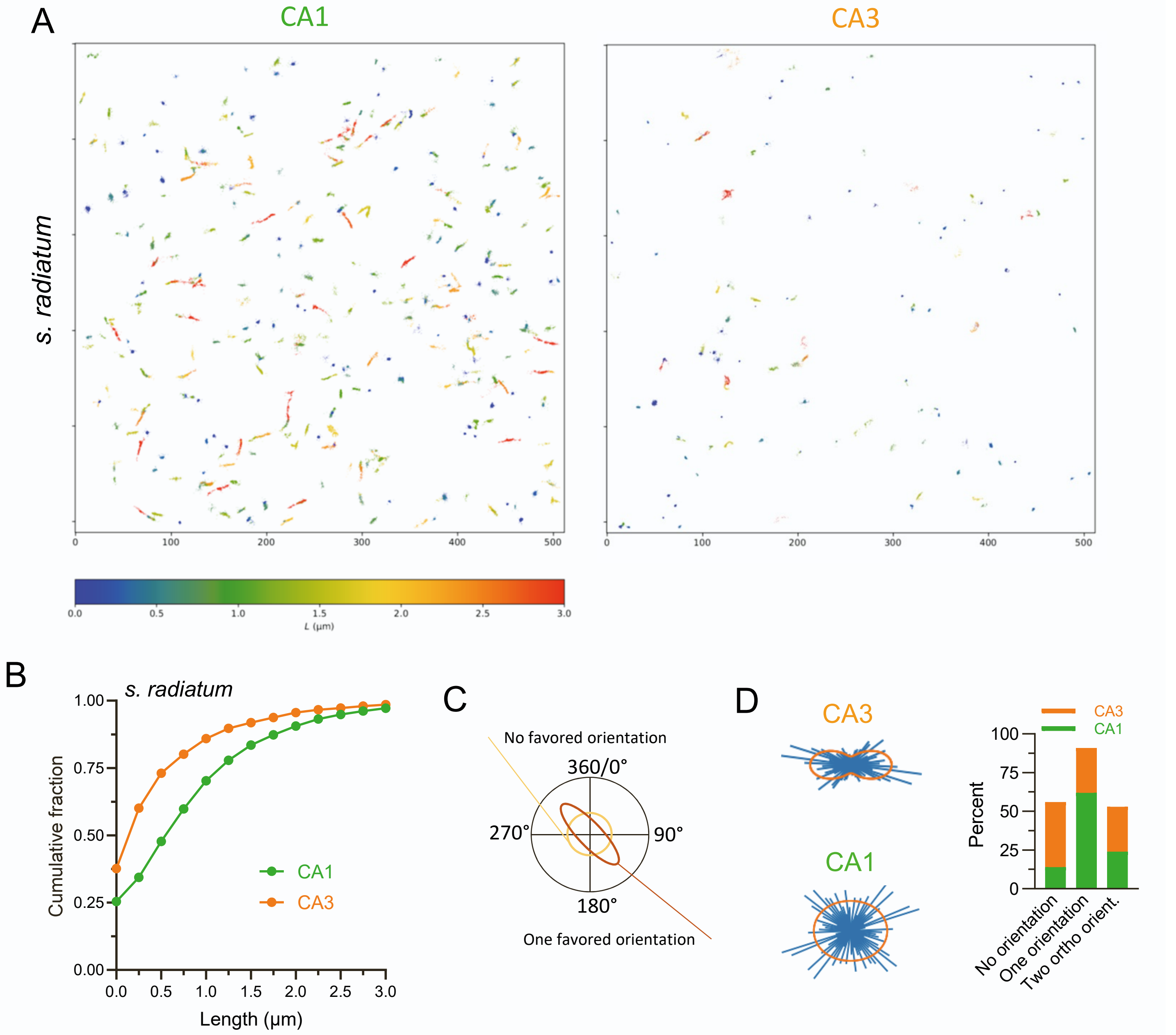

Figure S7. QD-based objects measurements (Related to Figure 1)

(A) Representative images showing length variations for s. *radiatum* in CA1 (left panel) and CA3 (right panel) areas using a QD-based cloud points analysis. Color-graded scale bar.

(B) Cumulative fractions plot of measured lengths for s. radiatum in CA3 and CA1 areas using a QD-based cloud point analysis.

Suppl. Figure 8

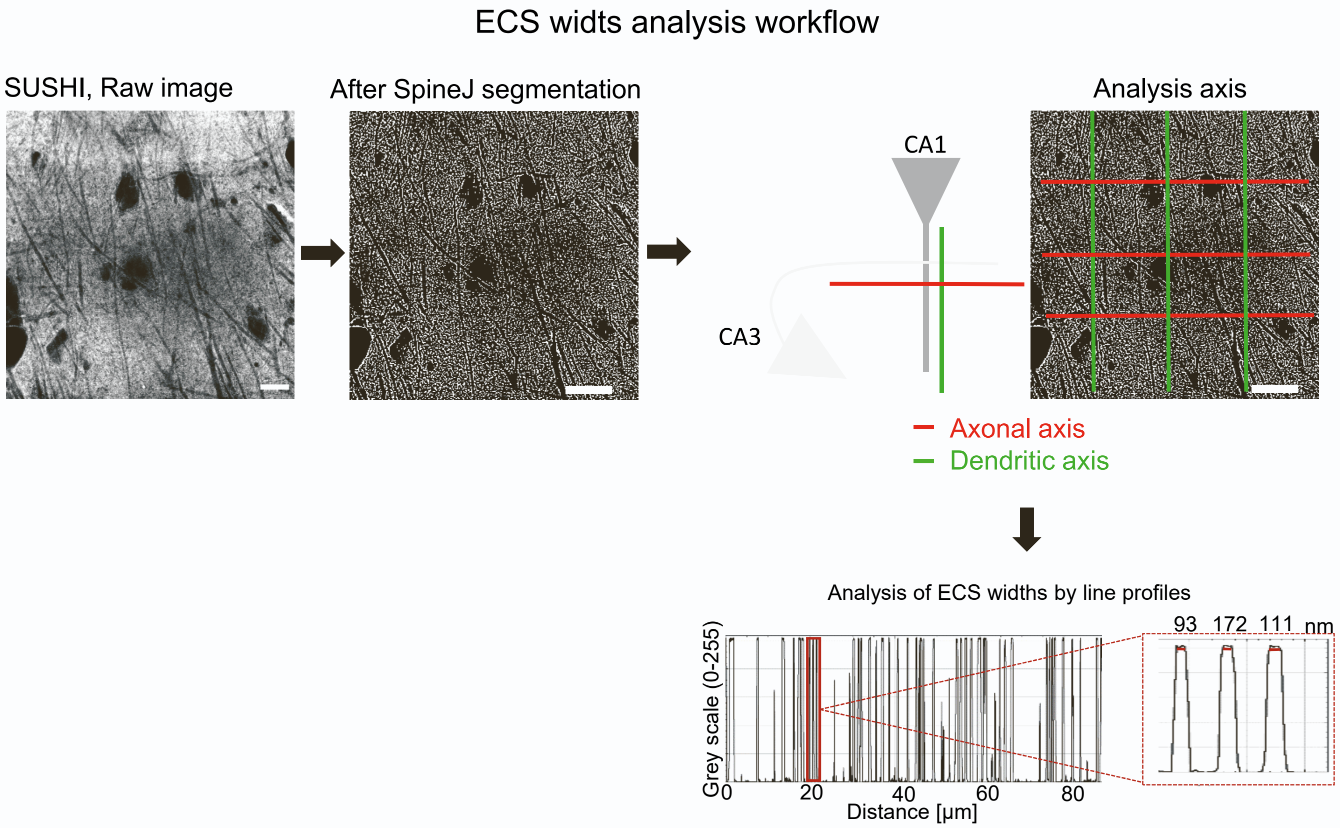

Figure S8. SUSHI-based ECS width analysis workflow (Related to Figure 3)
ECS widths analysis workflow. *Left panel*, Raw SUSHI image showing the ECS (white) and cellular elements (black) for a section of *s. radiatum* in the CA1 area (scale bar = 10 µm). *Middle panel*, A wavelet – based algorithm is used to binarize the raw SUSHI images. *Right panel*, Simplistic drawing of the CA3-CA1 connectivity, highliting the dendritic (green) and axonal (red) axis in the CA1 *s. radiatum*. Scanlines (line profiles) are drawn following the dendritic axis (i.e., paralell to the dendrites, green) and axonal axis (i.e., parallel to Schaffer collateral axons, red). *Low panel*: The peaks detected along the scanlines are measured to extract the ECS width on each detection, and then these values are plotted according their sizes.

Suppl. Figure 9

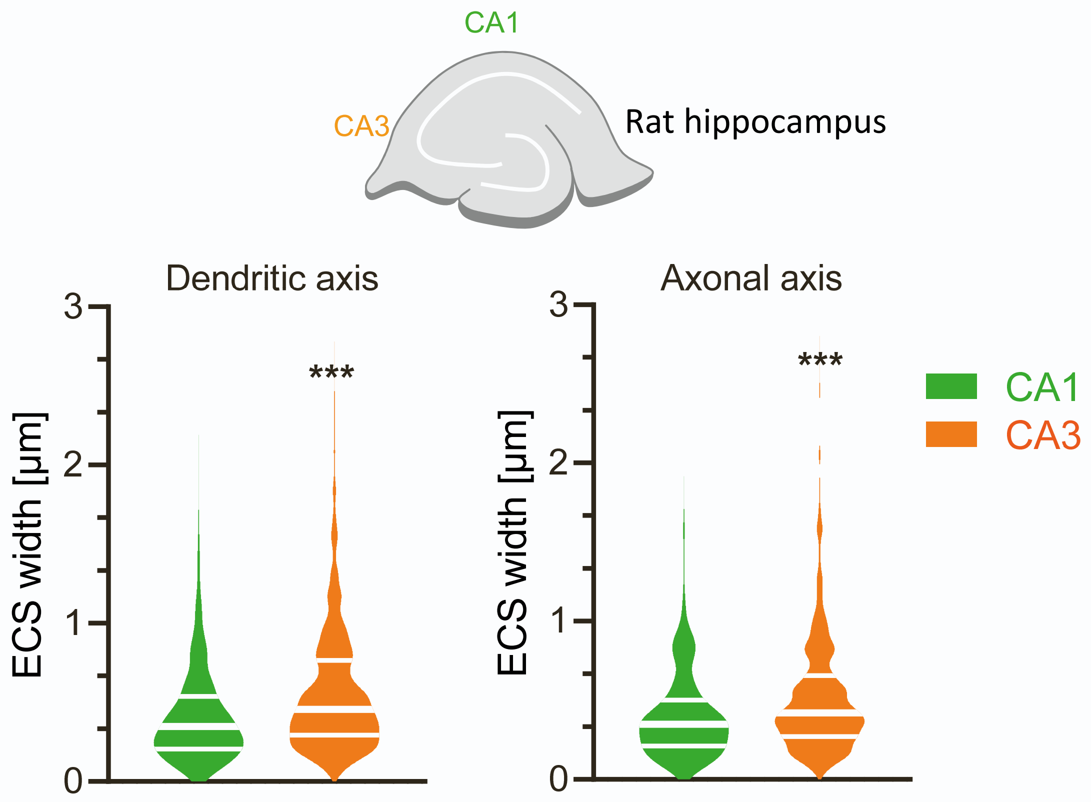

Figure S9. SUSHI-based exploration of the rat CA1 and CA3 areas (Related to Figure 3)
(A) Schematic representation of a rat hippocampal slice depicting CA3 and CA1 areas.
(B) ECS widths plots for *s. radiatum* in CA1 and CA3 areas for the dendritic axis (i.e., parallel to the dendrites) and axonal axis (i.e., parallel to Schaffer collateral axons).

Suppl. Figure 10

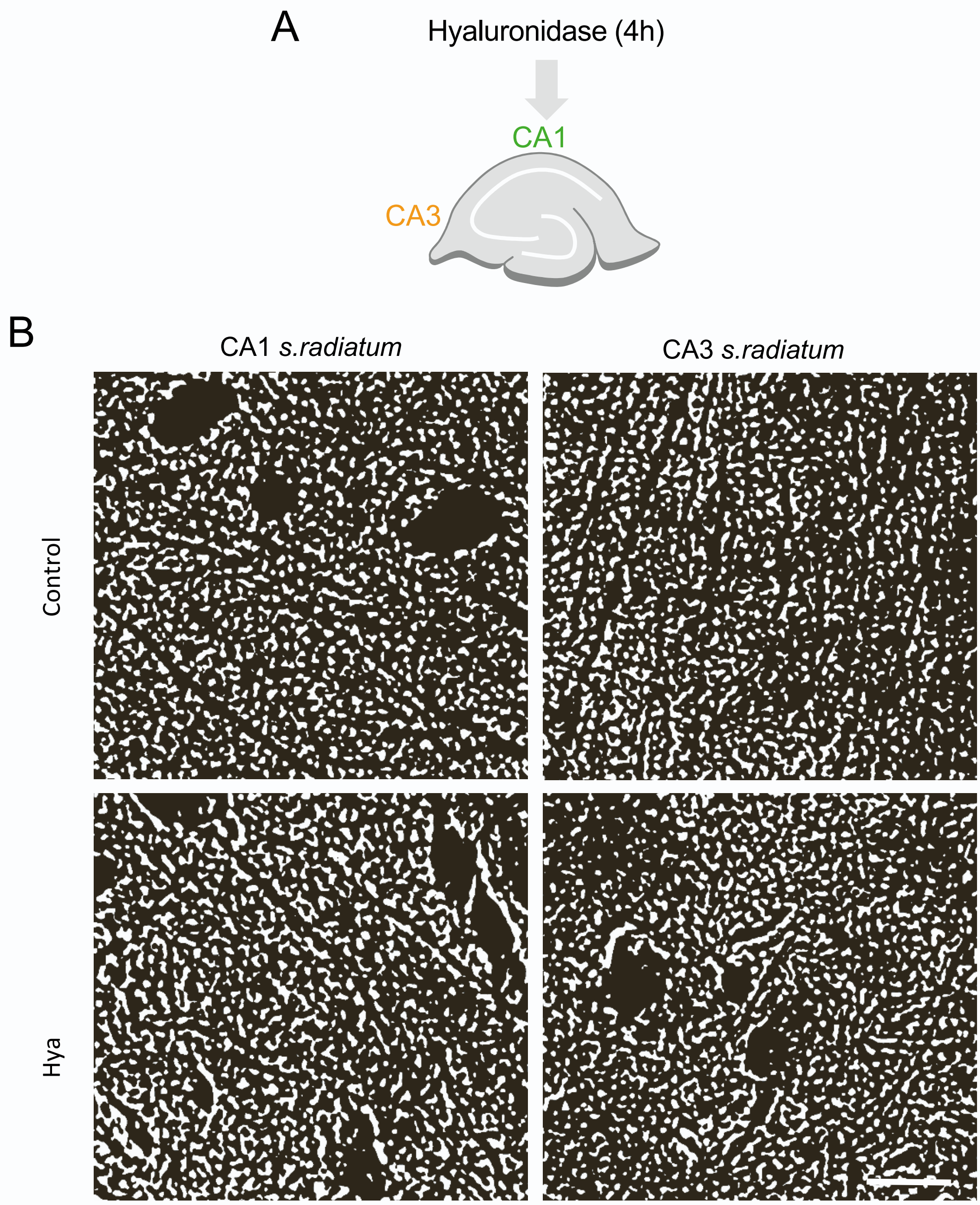

Figure S10. SUSHI-based imaging of CA1 and CA3 areas exposed to hyaluronidase (Related to Figure 3)
(A) Schematic representation of a rat hippocampal slice depicting CA3 and CA1 areas exposed to 4h treatment with hyaluronidase.
(B) Raw SUSHI images showing the ECS (white) for a section of *s. radiatum* in the CA1 and CA3 areas exposed to hyaluronidase (scale bar = 20 µm).
